# Tourism image perception of living historical and cultural neighborhoods under the HSSPR framework: A comparative study of three cases in Yunnan, China

**DOI:** 10.1371/journal.pone.0355218

**Published:** 2026-08-14

**Authors:** Nan Tao, Zhentao Yu, Qian Sun, Junyue Yang, Xiaoping Zhuang, Wen Duan

**Affiliations:** 1 School of Architecture and Urban Planning, Kunming University of Science and Technology, Kunming, China; 2 Kunming Municipal Center of Land Consolidation and Rehabilitation, Kunming, China; Sichuan Agricultural University, CHINA

## Abstract

Living historical and cultural neighborhoods (HCNs) integrate heritage conservation, community life, and tourism functions. Their tourism image is not shaped solely by historical resources or commercial provision, but is dynamically formed through interactions among material space, everyday settings, and visitor experience. Existing destination image studies still provide a limited explanation of this spatially situated process. Focusing on three issues—the construction of an analytical framework and evaluation system, the identification of perceptual differences under different degrees of tourism development, and the diagnosis of bottlenecks hindering image optimization—this study examines three living HCNs in Yunnan, China: Longwei Pass in Dali, Santai Mountain and its surrounding historic area in Chenggong Old Town, Kunming, and the Ming Walled Town–Qing Old County Seat area in Tonghai, Yuxi. It proposes an exploratory HSSPR framework (Human–Space–Setting–Place–Realm) and integrates UGC text analysis, TextCNN-based sentiment recognition, and adaptive importance–performance analysis (IPA) to identify elements of visitor perception, measure sentiment tendencies, and assess optimization priorities. The results show that: (1) the HSSPR framework helps translate fragmented UGC expressions into four hierarchical dimensions: basic spatial support, recreational and consumption experience, historical landscape cognition, and emotional meaning and resonance; (2) the three sites all show perceptual features in which historical continuity, spatial environment, and everyday life are interwoven, but differ in tourism development intensity and spatial organization. Longwei Pass represents a living neighborhood in which mountainous border-pass streets and alleys, and everyday marketplace life are continuously generated through low-intensity, largely spontaneous leisure consumption. Santai Mountain Historic Area is a composite historic area where the park, surrounding communities, and religious, cultural, and educational nodes coexist and interact amid limited tourism development. Ming-Qing Tonghai Old Town represents an established ancient-town neighborhood in which the mountain–water ancient-town structure, Confucian Temple culture, and local life are integrally coupled under moderate cultural-tourism renewal; (3) historical and cultural relics are a shared strength across the three sites, whereas selected elements of basic spatial support, recreational and consumption experience, natural ecological landscape, and physical and mental experience show high-attention–low-performance mismatches across the cases. These findings indicate that the main bottlenecks hindering tourism image enhancement in living HCNs lie not in insufficient resource appeal but in the inadequate transformation of resources, local life, and experience. This study extends research on tourism image perception in living HCNs from a spatialized and contextualized perspective and provides an operational analytical reference for living heritage conservation, service improvement, and image formation in similar neighborhoods.

## 1. Introduction

Historical and cultural neighborhoods (HCNs) are important spatial carriers of urban historical continuity, local memory, and everyday life. They are also among the socio-spatial fields in which tensions between cultural heritage conservation and tourism use most frequently arise [1,2]. Unlike enclosed scenic areas oriented primarily toward sightseeing consumption, living HCNs remain embedded in residents’ daily routines, community functions, and local social relations. Their spatial value is reflected not only in architectural remains, street-and-alley patterns, and historic landscapes but also in ongoing local life, social interaction, and everyday consumption [3]. The tourism image of living HCNs is therefore not shaped solely by heritage resources, commercial formats, or visual symbols; rather, it emerges from the ongoing interplay among heritage spaces, community life, and tourism experiences. In recent years, the growth of cultural tourism and the advancement of urban renewal have increased the visibility and spatial vitality of HCNs. At the same time, these areas have experienced commercial expansion, the weakening of local distinctiveness, superficial cultural representation, and the homogenization of tourism experiences [4]. How to construct a tourism image that can be recognized, understood, and identified with by visitors while sustaining heritage authenticity and the continuity of community life has become a key issue for the sustainable conservation and use, tourism planning, and heritage management of HCNs.

Perception of tourism image offers an important perspective for understanding these issues. Previous studies have examined destination image [5, 6], conservation and renewal of HCNs [7], tourism experience, and tourist behavior [8,9], providing a basis for understanding the conservation and renewal of HCNs. However, for HCNs that still retain everyday living functions, existing research has not sufficiently explained the hierarchical formation of tourism image within the intertwined context of “heritage attributes–everyday life–tourism experience.” Limited attention has also been paid to the commonalities and differences in the perceptual structures of different types of HCNs within the same region.

Therefore, this study selects three living HCNs in Yunnan, China, as empirical cases: Longwei Pass Living Historical and Cultural Neighborhood in Dali, Santai Mountain and Surrounding Living Historic Area in Chenggong Old Town, Kunming, and the Ming Defensive Garrison Town–Qing Old County Seat Living Historical and Cultural Neighborhood in Tonghai, Yuxi. All three sites combine historical remains, traditional streets and alleys, community life, and tourism consumption, and none has been fully transformed into a highly commercialized scenic area or social media “check-in” hotspot. At the same time, they represent different types of living HCNs: a border-pass mountainous living type, a park–community composite type, and an established ancient-town living type. These differences provide a basis for comparative analysis. This study develops an evaluation framework for tourism image perception in living HCNs and integrates UGC text analysis, sentiment recognition, and IPA analysis to identify visitors’ perceptual elements, emotional evaluations, and optimization priorities. Specifically, the study addresses the following three research questions:

1. How can an exploratory HSSPR analytical framework and evaluation system be developed to assess tourism image perception in living HCNs?
2. Based on the commonalities and differences among the empirical cases, what perceptual characteristics of tourism image distinguish living HCNs from single-function sightseeing destinations, and how are these characteristics formed?
3. Which perceptual elements are more strongly favored by visitors in HCNs, and what key deficiencies constrain the enhancement of their tourism image?

This study aims to provide a hierarchical, spatial, and context-sensitive explanatory approach for research on tourism image in living HCNs. Theoretically, it incorporates tourism image perception, spatial experience, and the generation of place meaning into a single analytical framework. Methodologically, it explores the integration of UGC text analysis, sentiment recognition, and IPA diagnosis. Practically, it offers references for tourism planning, service optimization, heritage interpretation, and place image management in living HCNs.

## 2. Research progress and theoretical framework construction

### 2.1. Research progress

#### 2.1.1. Research on destination image.

Destination image (DI) is generally understood as the comprehensive mental representation that visitors form of a destination’s resources, environment, services, and experiences. Since Hunt introduced the destination image concept into tourism studies [10], research in this field has gradually developed an analytical approach centered on cognitive image, affective image, and behavioral intention [11]. Echtner and Ritchie extended the measurement of destination image through dimensions such as attribute–holistic, functional–psychological, and common–unique components [12], while Baloglu and McCleary proposed the “cognitive–affective–overall image” model [13]. These studies indicate that destination image research is well-equipped with multidimensional frameworks; existing theories can effectively explain visitors’ attribute cognition, emotional evaluation, and behavioral tendencies toward general destinations.

However, classical DI models have mainly been developed for ordinary natural scenic areas or macroscale urban destinations [14,15]. Their systemic frameworks are largely built upon visitors’ psychological processes and evaluations of destination attributes, frequently overlooking the specific spatial structures, social activities, and local cultural contexts that anchor these perceptions. For living HCNs, the object of visitor perception is not merely an isolated attraction, commercial service, or cultural symbol. Rather, it is a composite field shaped by heritage spaces, community life, recreational consumption, local memory, and symbolic meanings. If the “cognitive–affective–conative” framework is rigidly applied, the tourism image of HCNs risks being simplified into mere attribute evaluation, emotional tendency, or behavioral outcome. Consequently, this framework fails to adequately explain how visitors’ perceptions are triggered and organized within specific spatial hierarchies and living contexts, and how they gradually evolve into emotional identification and meaningful resonance. Research on the tourism image of living HCNs, therefore needs to build on existing DI theory while incorporating the analytical perspectives of spatial hierarchy, situated experience, and meaning generation.

#### 2.1.2. UGC-based research on tourism image perception and its limitations.

With the development of online travel platforms and social media, user-generated content (UGC) has become an important data source for research on the perception of tourism image [16]. Compared with questionnaires and interviews, UGC offers several advantages, including more natural expression, stronger contextuality, and broader coverage. It can capture visitors’ spontaneous attention and emotional responses during actual travel experiences and subsequent online sharing. In recent years, related studies have moved beyond word-frequency statistics and content analysis to include quantitative approaches such as text mining, semantic network analysis, sentiment analysis, and IPA [17–20].

However, the use of UGC does not in itself ensure greater explanatory depth. Some studies remain confined to a largely descriptive level of “high-frequency word extraction–theme classification–sentiment judgment,” treating transportation, architecture, commerce, folk customs, and emotions as parallel indicators. This approach often fails to adequately explain the spatial embeddedness, hierarchical relationships, and planning implications of different perceptual elements. Without the guidance of a systematic hierarchical theory, UGC analysis can easily be reduced to a mere enumeration of high-frequency words and fail to reveal the structural interactions among physical spaces, social settings, cultural contexts, and conceptual meanings. Therefore, the use of UGC in this study does not seek to highlight the novelty of text mining itself. Rather, it aims to integrate fragmented visitor perceptions from UGC into a codable and comparable hierarchical analytical framework, thereby providing a basis for the subsequent construction of the evaluation system and multi-case comparison.

#### 2.1.3. Specificity of tourism perception in living HCNs.

Unlike highly scenic area-oriented tourism spaces, living HCNs retain the everyday routines of long-term residents, local community relations, and living cultural heritage. Their tourism image is not determined solely by visual symbols or commercial formats, but is dynamically generated through multidimensional interactions among “human–space–activity–meaning” [21]. Existing research on HCNs has generally followed three lines of inquiry. The first focuses on conserving street fabric, revitalizing public spaces, and organizing visitor routes, emphasizing the preservation, renewal, and functional efficiency of historic spaces [22–25]. The second examines commercial restructuring, heritage interpretation, and authenticity-based experiences, with attention to how tourism development affects local cultural representation and everyday neighborhood life [26,27]. The third explains tourism experience outcomes from the perspectives of tourist satisfaction, place attachment, and behavioral intention [8,28]. These studies provide an important basis for understanding the conservation and use of HCNs. However, the current literature still lacks an explanatory framework that systematically integrates spatial hierarchy, living context, visitor perception, and the mechanisms underlying the formation of tourism image in HCNs. Comparative analysis across different types of living HCNs also remains largely underexplored.

### 2.2. Construction of the progressive HSSPR analytical framework

(1) Complementarity of the theoretical foundations: from spatial recognition to resonance of meaning

To address the insufficient linkage among spatial hierarchy, social context, and visitor perception in research on the tourism image of living HCNs, this study builds on the composite “heritage–life” attributes of HCNs and integrates spatial cognition theory [29,30], phenomenological space theory [31], scene theory [32], sense of place theory [33], and Lefebvre’s spatial triad [34,35]. On this basis, it proposes an exploratory HSSPR (Human–Space–Setting–Place–Realm) analytical framework. This framework is not intended to replace classical destination image models or to demonstrate a strict causal mechanism. Instead, it is designed to organize and interpret fragmented visitor perceptions expressed in UGC and to provide a hierarchical coding basis for perceptual elements.

These theories correspond to different stages in the formation of visitor perception. Spatial cognition theory emphasizes how individuals identify and organize spatial elements such as paths, nodes, edges, districts, and landmarks. It therefore provides a basis for explaining how visitors form basic cognition through streets and alleys, buildings, facilities, and the surrounding environment. Phenomenological space theory focuses on bodily presence, sensory experience, and everyday practice [36], while scene theory further emphasizes the configuration of “human–activity–space–atmosphere” [37]. Together, these perspectives explain how visitors move from “viewing space” to “experiencing settings” through street strolling, food consumption, community observation, and folk activities. Sense of place theory emphasizes that space is transformed into “place” with identity-related meanings through memory, emotion, and value attribution [38]. It supports the interpretation of visitors’ emotional responses to local culture, everyday atmosphere, and authenticity-based experiences. Lefebvre’s spatial triad further frames HCNs as the outcome of the continuous interaction among social practices, cultural representations, and everyday life [39]. It helps explain how historical narratives, collective memory, belief systems, and value symbols may further evoke resonance of meaning at the spiritual level.

Accordingly, this study conceptualizes tourism image perception in living HCNs as a progressive process that moves from initial spatial recognition to deeper meaning. Spatial cognition theory addresses how space is recognized; phenomenological space theory and scene theory explain how space is experienced; sense of place theory clarifies how space engenders identification; and the theory of the production of space explains how space is endowed with spiritual meaning. This theoretical chain provides the foundation for the HSSPR framework. It organizes spatial recognition, situated experience, place identity, and resonance of meaning into an explanatory sequence that can be used for text coding and case comparison, thereby distinguishing it from classical destination image models that primarily explain image formation through tourists’ psychological dimensions.

(2) Construction of the HSSPR framework: the progressive formation mechanism from space to context and meaning

**Fig 1:**
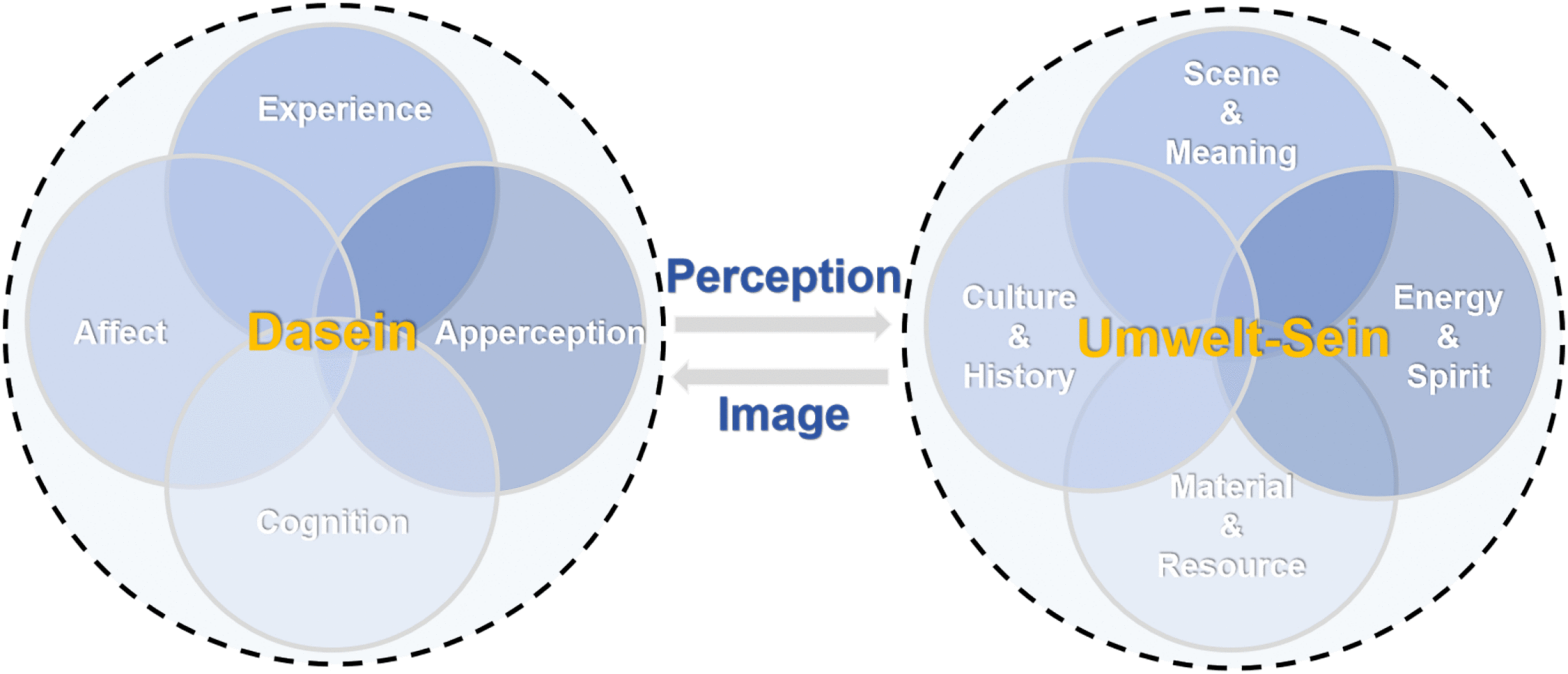
Diagram of the “Human-Field” interaction in HCNs.

**Fig 2:**
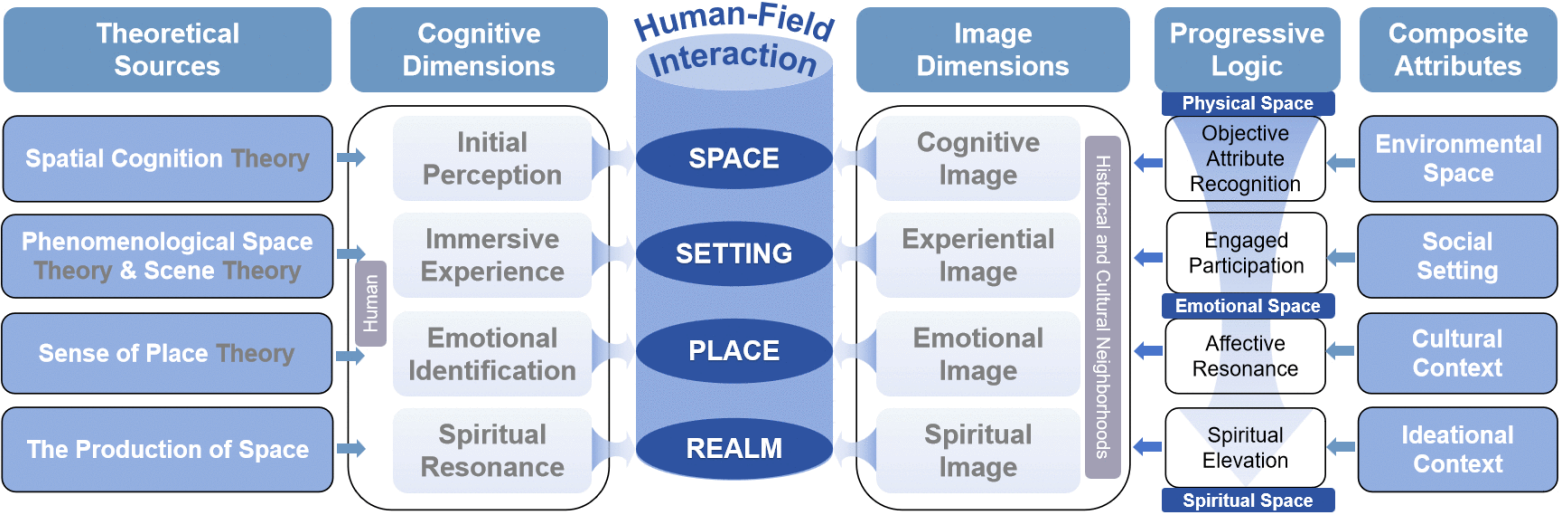
The HSSPR hierarchical framework of tourism image perception.

The HSSPR framework takes the interaction between the human subject (Dasein) and the field system (Umwelt-Sein) as its theoretical point of departure (Fig 1). It divides the objects of tourism image perception in living HCNs and their corresponding image dimensions into four progressively related layers (Fig 2). The framework does not assume a one-way linear causality. Rather, it is intended to explain how visitor perception moves from cognition of the external environment toward experiential evaluation and resonance of meaning. On this basis, this study classifies perceptual objects and their image mappings into four layers: Space, Setting, Place, and Realm. These layers are not closed or linear causal stages, but analytical tools for organizing visitor perception, identifying the hierarchy of perceptual elements, and comparing differences across cases.

(1) Space refers to the environmental space layer, namely the material spatial foundation of the neighborhood that visitors can directly identify. It includes transportation facilities, public services, signage systems, historical remains, and related elements. This layer constitutes visitors’ initial perception after entering the neighborhood and mainly corresponds to the cognitive image in tourism image formation. It emphasizes visitors’ intuitive judgments of spatial order, environmental conditions, and infrastructure.
(2) Setting refers to the social setting layer, which denotes the immersive experiences formed through daily leisure, dining, and entertainment, commercial consumption, and social interaction within the neighborhood. The distinctiveness of living HCNs lies in the fact that heritage spaces are not detached from residents’ everyday lives; instead, they form dynamic settings alongside local consumption, community activities, and tourist participation. This layer marks a shift in tourism image from static spatial cognition toward process-oriented experience and primarily corresponds to the experiential image.
(3) Place refers to the cultural context layer, which denotes the cultural understanding and emotional identification formed through historical narratives, traditional cultural relics, collective memory, place attachment, and sense of belonging. Its core lies not merely in identifying historical resources themselves, but in understanding how spatial patterns, cultural continuity, and everyday practices jointly constitute meaningful places with a sense of locality. This layer mainly corresponds to the emotional image, reflecting visitors’ judgments of local culture, everyday atmosphere, and authenticity-based experiences.
(4) Realm refers to the ideational context layer, which denotes the spiritual resonance formed on the basis of the visiting experience, including belief systems, local spirit, value symbols, and spiritual resonance. At this stage, the image of the neighborhood is no longer limited to visible resources or immediate experience. It is further transformed into a subjectively constructed form of spiritual perception, mainly corresponding to the spiritual image and reflecting visitors’ comprehensive judgments of local values, memory sustenance, and intentions to revisit or recommend.

Based on these four layers, this study translates perceptions of tourism image in living HCNs into four codable dimensions: basic spatial support, recreational and consumption experiences, historical landscape cognition, and emotional meaning and resonance. These dimensions are then used to construct the subsequent system of 16 perceptual elements. This approach helps avoid the simplistic juxtaposition of heterogeneous UGC expressions related to space, commercial formats, culture, and emotion. It also provides a unified framework for multi-case comparison.

#### 2.2.1. Structural differences between the HSSPR framework and classical models.

Compared with classical DI models, the HSSPR framework differs primarily in three aspects: explanatory perspective, hierarchical logic, and data organization. First, classical models tend to emphasize changes in tourists’ psychological processes, whereas the HSSPR framework re-embeds psychological perception within the physical and social spatial hierarchy of HCNs, highlighting the spatial embeddedness and contextual activation of tourism image formation. Second, classical models often focus on the linear influence of attribute evaluation on behavioral intention, whereas HSSPR is designed specifically for living HCNs and can capture and explain the progressive transformation along the sequence of “material space–setting participation–cultural empathy–spiritual resonance”. Third, in response to the highly fragmented nature of perceptual content in UGC texts, the HSSPR framework uses the four layers of Space, Setting, Place, and Realm to provide a classification and coding system with a rigorous hierarchical logic, as well as a quantitative diagnostic pathway for large-scale unstructured online texts. This helps avoid the simplistic juxtaposition of heterogeneous perceptual content.

In summary, HSSPR can be operationalized as a codable, comparable, and preliminarily testable analytical tool. This study uses it to construct an analytical and evaluation framework for tourism image perception in living HCNs. By comparing the word-frequency, semantic, and sentiment characteristics of UGC across the three cases, the study identifies shared perceptual features and type-specific differences across neighborhoods, and uses IPA quadrant analysis to diagnose the strengths and key constraints that limit tourism image enhancement.

## 3. Materials and methods

### 3.1. Overview of the study sites

**Fig 3:**
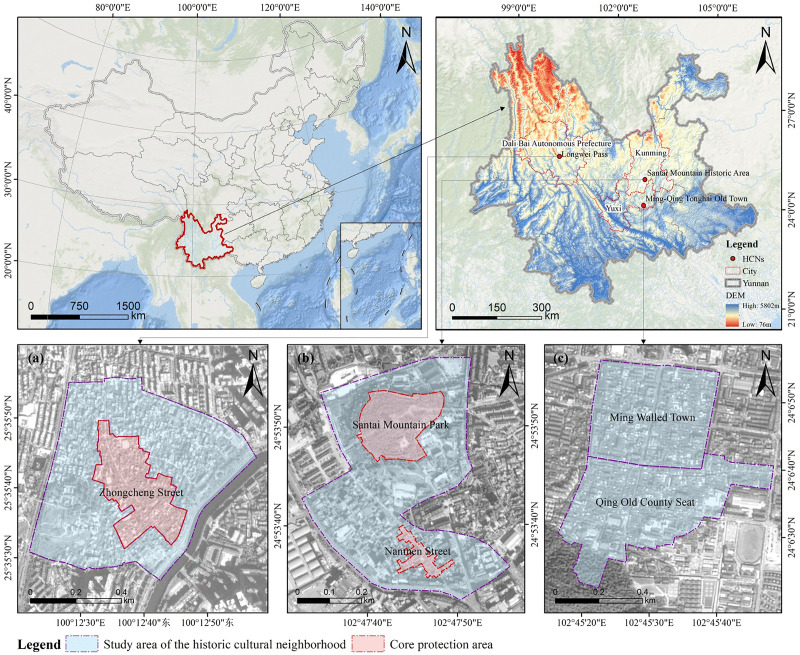
Location of the study sites.

This study selected three living HCNs in Yunnan Province, China, that still retain everyday living functions and represent complementary types: Longwei Pass, Santai Mountain Historic Area, and Ming-Qing Tonghai Old Town. All three sites combine historical remains, traditional streets and alleys, community life, and local consumption, alongside varying degrees of tourism development. However, they differ in their historical formation mechanisms, spatial organization, forms of community life, and levels of tourism development, making them suitable for a multi-case comparison of tourism image perception in living HCNs (Fig 3).

Longwei Pass is located in the Xiaguan area of Dali City, at the foot of Cangshan Mountain and on the northern bank of the Xi’er River. It once served as the southern gateway of the Nanzhao and Dali Kingdoms and as an important passage along the Tea Horse Ancient Road. The neighborhood retains the remains of the historic pass, ancient streets and alleys, traditional courtyards, and the marketplace life of old Xiaguan. Its mountain–water setting remains closely interwoven with neighborhood life, while tourism activities mainly involve strolling through streets and alleys, local dining, and light consumption. It can therefore be characterized as a border-pass mountainous living HCN shaped by low-intensity and largely spontaneous leisure consumption.

Santai Mountain Historic Area is located in Chenggong Old Town, Kunming. The area centers on Santai Mountain Park as its primary public recreational space, is surrounded by local communities, and incorporates religious, educational, and cultural resources such as the Confucian Temple, Santai Temple, Kuige Pavilion, and Bing Xin’s Molu. Influenced by the development of Chenggong New Area, the traditional urban fabric is interwoven with modern urban space. Tourism development remains limited, and visitors’ perceptions are shaped mainly by park recreation, community life, and cultural-educational nodes. It can therefore be characterized as a park–community composite historic area under limited tourism development.

Ming-Qing Tonghai Old Town is located in the historic urban area of Tonghai County, Yuxi City, and consists of two HCNs: the Ming defensive garrison town and the Qing old county seat. With Xiushan Mountain to the south and Qilu Lake to the north, the area retains a relatively complete Ming–Qing ancient-town pattern, Confucian Temple culture, traditional streets and alleys, and local living spaces. It presents an integrated coupling of mountain–water spatial structure, ritual culture, and community life, and can therefore be characterized as a living HCN rooted in a historic urban administrative structure and undergoing moderate culture-and-tourism-oriented renewal. Overall, the three sites respectively reflect differences in continuity, complexity, and integrity, providing a case basis for the subsequent comparison of UGC-based perceptions.

### 3.2. Research methodology

#### 3.2.1. Network text analysis and deep-learning-based sentiment computation workflow.

Network text analysis can extract visitors’ perceptual themes, sentiment tendencies, and evaluative elements from user-generated content (UGC), making it suitable for identifying the perceptual structure of tourism image in living HCNs. Compared with traditional questionnaires, UGC provides a larger sample size and more closely reflects visitors’ spontaneous expressions. It can therefore supplement the analysis by capturing immediate feelings, spatial evaluations, and emotional feedback during tourism experiences. Based on the hierarchical HSSPR framework, this study developed an analytical workflow consisting of “data collection–text cleaning–sentiment recognition–element matching–IPA diagnosis”. First, multi-platform online data were collected for the three cases: Longwei Pass, Santai Mountain Historic Area, and Ming-Qing Tonghai Old Town. Second, the raw texts were processed through deduplication, word segmentation, stop-word filtering, removal of irrelevant information, and expression standardization, generating a valid analytical corpus. Third, by combining sentiment recognition results with a keyword feature lexicon, visitor comments were mapped onto specific perceptual elements under the HSSPR framework. Finally, the frequency of element mentions was used to represent attention salience, while sentiment evaluation results were used to represent performance. These indicators were then incorporated into the adaptive IPA model to identify perceptual advantages, experiential deficiencies, and optimization priorities across the different neighborhoods.

In the sentiment recognition stage, given that short online reviews are inherently characterized by colloquial language, fragmented expression, and strong context dependence, relying solely on sentiment dictionaries or traditional machine-learning methods may be inadequate due to insufficient contextual adaptability and a limited ability to identify implicit emotions. Therefore, this study built a text convolutional neural network (TextCNN) model for sentiment tendency recognition using Python and the PyTorch framework. To improve the model’s adaptability to the context of tourism reviews on HCNs, the training corpus was drawn from historic towns or neighborhoods in Yunnan that share similarities with the three empirical cases in expressions related to historical remains, traditional streets and alleys, local life, and tourism experience. These sources included Heijing Ancient Town, Weishan Ancient City, Shaxi Ancient Town, and Lijiang Ancient City. The training corpus covered UGC from online platform reviews, Xiaohongshu posts, and related comments, encompassing common expression scenarios such as historic sites, street-and-alley spaces, local dining, commercial consumption, transportation services, physical and mental experiences, and recommendation intentions.

On this basis, model training followed a weakly supervised learning procedure of “rule-based annotation–manual review–TextCNN”. First, review texts were preliminarily labeled as positive, neutral, or negative according to rules based on sentiment dictionaries, degree adverbs, negation words, and contrastive structures. The preliminary labels were then manually reviewed, with particular attention to correcting misclassified samples involving irony, mixed emotions, very short sentences, and ambiguous sentiment expressions. This process produced a deduplicated labeled dataset of 7,532 records, including 4,035 positive, 2,766 neutral, and 731 negative samples. To test the reliability and robustness of the model recognition results, the labeled dataset was divided through stratified sampling into a training set of 6,025 records, a validation set of 753 records, and a test set of 754 records, while maintaining the same proportional distribution of the three sentiment labels. The model used character-level encoded texts as input. The maximum text length was set to 160, and the character vocabulary size was 3,618. The main parameters included an embedding dimension of 64, 64 convolution kernels, convolution kernel sizes of [2–5], a batch size of 64, and 10 training epochs. The TextCNN model extracted local semantic combination features from short texts and used a Softmax layer to produce the probability distribution over sentiment categories. The test-set results showed a classification accuracy of 86.34%, a macro-precision of 80.26%, a macro-recall of 78.28%, and a macro-averaged F1 score of 0.7918, indicating that the model performed well in identifying sentiment in short tourism reviews related to HCNs. The trained model was then applied to batch sentiment recognition of comments from the three study sites, providing a basis for subsequent performance measurement and IPA analysis.

#### 3.2.2. Importance–performance analysis.

To incorporate perceptual attention and sentiment evaluation from online texts into a unified diagnostic framework, this study adapts the traditional importance–performance analysis (IPA). The frequency with which an element is mentioned in online reviews is not fully equivalent to subjective importance measured through questionnaires. Therefore, the IPA results in this study are used primarily to identify mismatches between “attention” and “experience” in tourists’ online expressions, rather than to establish strict causal relationships. In this study, “importance” is treated as a proxy indicator of attention salience in tourists’ perceptions. “Performance”—conceptualized here as experiential performance—is calculated from the sentiment probability distribution generated by the TextCNN model, serving to represent tourists’ experiential feedback on the relevant perceptual elements.

First, the importance score of each perceptual element was calculated. For a given case, if the i-th review was matched to the n-th perceptual element, then $x_{i,n}$=1; otherwise, $x_{i,n}$=0. The occurrence frequency $f_{n}$ of the n-th perceptual element was calculated as follows:


$$\begin{array}{c} \begin{array}{c} f_{n}=\sum_{i=\text{1}}^{m}x_{i,n} \end{array} \end{array}$$ (1)

Where $f_{n}$ denotes the occurrence frequency of the n-th perceptual element in a given case, and m denotes the number of valid experiential evaluation texts in that case. The proportion of the occurrence frequency of this element to the total occurrence frequency of all perceptual elements was then used as its importance score$\;I_{n}$:


$$\begin{array}{c} \begin{array}{c} I_{n}=\frac{f_{n}}{\sum_{k=\text{1}}^{N}f_{k}}\times \text{1}\text{0}\text{0}\% \end{array} \end{array}$$ (2)

Where $I_{n}\;$denotes the importance score of the n-th perceptual element, and N denotes the total number of core perceptual elements extracted through text mining and grounded coding. The denominator $\sum_{n=\text{1}}^{N}f_{k}$ represents the total occurrence frequency of all perceptual elements in the given case.

Second, the performance score of each perceptual element was calculated. To avoid obscuring intensity differences within the same sentiment category through the discrete assignment of “positive = 5, neutral = 3, negative = 1”, this study constructed a continuous sentiment performance score for each review based on the three-class sentiment probabilities output by the TextCNN model:


$$\begin{array}{c} \begin{array}{c} S_{i}=\text{5}p_{i}^{pos}+\text{3}p_{i}^{neu}+\text{1}p_{i}^{neg} \end{array} \end{array}$$ (3)

where $S_{i}$ denotes the sentiment performance score of the i-th review; $p_{i}^{pos},p_{i}^{neu}$and $p_{i}^{neg}$ denote the probabilities of positive, neutral, and negative sentiment output by the TextCNN model, respectively, with their sum equal to 1. The performance score of the n-th perceptual element was represented by the mean sentiment performance score of all reviews matched to that element:


$$\begin{array}{c} \begin{array}{c} P_{n}=\frac{\sum_{i=\text{1}}^{m}x_{i,n}S_{i}}{\sum_{i=\text{1}}^{m}x_{i,n}} \end{array} \end{array}$$ (4)

Finally, IPA quadrants were constructed using a dynamic mean-splitting method. The mean importance score $\bar{I}$ and mean performance score $\bar{P}$ of all perceptual elements in a given case were calculated and used as the dividing axes of the four-quadrant matrix:


$$\begin{array}{c} \begin{array}{c} \bar{I}=\frac{\text{1}}{N}\sum_{n=\text{1}}^{N}I_{n} \end{array} \end{array}$$ (5)

$$\begin{array}{c} \begin{array}{c} \bar{P}=\frac{\text{1}}{N}\sum_{n=\text{1}}^{N}P_{n} \end{array} \end{array}$$ (6)

According to the position of each element’s coordinates (${\;I}_{n},P_{n}$) relative to the mean lines, the elements were classified into four quadrants: maintain advantages, priority improvement, moderate maintenance, and low priority:


$$\begin{array}{c} \begin{array}{c} Q_{n}=\left\{ \begin{array}{c} Maintain\;advantages,{\;I}_{n}\geq \bar{I},\;P_{n}\geq \bar{P}\\ Priority\;improvement,\;I_{n}\geq \bar{I},\;P_{n}<\bar{P}\\ Moderate\;maintenance,I_{n}<\bar{I},\;P_{n}\geq \bar{P}\\ Low\;priority,\;I_{n}<\bar{I},\;P_{n}<\bar{P} \end{array}\right. \end{array} \end{array}$$ (7)

Where $Q_{n}$ denotes the quadrant assignment of the n-th perceptual element. Given that online UGC texts generally show a concentration of positive sentiment and relatively high overall performance scores, this study used dynamic dual means rather than fixed midpoint values as the dividing lines. This approach allows the matching or mismatch between attention salience and experiential performance to be identified more effectively.

### 3.3. Data sources

#### 3.3.1. Multi-platform online data collection.

This study selected Longwei Pass, Santai Mountain Historic Area, and Ming-Qing Tonghai Old Town as empirical study sites. As the target users were mainly domestic Chinese tourists, online textual data were obtained from three major Chinese online platforms: Dianping (https://www.dianping.com), Ctrip (https://www.ctrip.com), and Xiaohongshu (https://www.xiaohongshu.com). These platforms are respectively characterized by consumer reviews, travel evaluations, and social media sharing, and thus can reflect visitors’ spatial experiences, consumption perceptions, and overall impressions of living HCNs from different perspectives.

Given the differences in data structure and textual presentation across platforms, this study adopted platform-specific collection strategies. Data from Ctrip were obtained using Python Requests to access publicly available web interfaces, through which comment text, posting time, user ratings, and related fields were collected. Data from Xiaohongshu were collected using Selenium-based automated web scraping, targeting post titles, body text, and multi-level comment texts. Data from Dianping were obtained using Reqable, a network protocol analysis tool, to parse publicly available review data from mobile H5 pages and WeChat mini-programs, extracting fields such as review text, posting time, and star ratings. To ensure proper and ethical use of data, only publicly available texts were collected, and no private communications or sensitive information were involved. User identifiers were used only for deduplication and sample validation and were excluded from subsequent statistical analyses.

After multi-platform collection and preliminary aggregation, a total of 4,219 initial online textual records were obtained. These included 1,619 records for Longwei Pass, 914 records for Santai Mountain Historic Area, and 1,686 records for Ming-Qing Tonghai Old Town. The volume of UGC was generally consistent with the degree of tourism development across the cases. The distribution of raw texts across platforms and cases is shown in Table 1.

**Table 1:** Number of raw online textual records collected by the platform.

Case Site	Longwei Pass	Santai Mountain Historic Area	Ming-Qing Tonghai Old Town	Total
**Dianping**	316	129	190	635
**Ctrip**	4	2	70	76
**Xiaohongshu**	1299	783	1426	3508
**Number of raw texts**	1619	914	1686	4219

#### 3.3.2. Text preprocessing and sample screening.

The raw corpus collected from multiple platforms contained inconsistencies in formatting, duplicate content, and a mixture of texts unrelated to tourism experiences. To ensure the reliability of subsequent sentiment recognition, perceptual-element coding, and IPA analysis, the raw texts underwent standardized preprocessing and sample screening.

First, analytical units were reconstructed, and formats were cleaned across platforms. For platforms such as Xiaohongshu, where posts may include titles, body text, and multi-level comments, relevant content was reorganized into independent textual units. Fields such as the original platform source, comment level, original row number, and comment text were retained to ensure data traceability. Python scripts incorporating regular expressions were then used to clean the texts by removing noise such as whitespace characters, web links, platform tags, garbled characters, and emojis without clear semantic meaning. Exact duplicates were also removed. After preprocessing, 3,800 textual records were obtained across the three cases, including 1,506 for Longwei Pass, 843 for Santai Mountain Historic Area, and 1,451 for Ming-Qing Tonghai Old Town.

Second, texts not associated with tourism experiences were classified and screened. Comment sections on platforms such as Xiaohongshu often contain information-seeking texts about locations, routes, or photo spots; social reply texts such as emojis, greetings, and expressions of thanks; as well as short texts with limited information and texts expressing only interest arousal. Although these texts may reflect online engagement, they usually lack substantive evaluations of historical landscapes, spatial environments, facilities and services, local life, or recreational experiences. If included directly in the analysis, they could artificially inflate the proportion of neutral texts and affect the interpretation of IPA results. Therefore, this study combined keyword matching with manual review to exclude texts unrelated to tourism experiences. Only texts that explicitly addressed perceptual content related to landscapes, services, transportation, culture, spatial atmosphere, consumption experience, or local life were retained as valid samples. A total of 2,540 valid experiential evaluation texts were finally included in the main sentiment analysis and perceptual-element matching, including 1,020 for Longwei Pass, 536 for Santai Mountain Historic Area, and 984 for Ming-Qing Tonghai Old Town. The detailed screening results are shown in Table 2.

**Table 2:** Results of text preprocessing and valid sample screening.

Category	Longwei Pass	Santai Mountain Historic Area	Ming-Qing Tonghai Old Town	Total
**Number of texts after preprocessing**	1506	843	1451	3800
**Experience evaluation texts**	1020	536	984	2540
**Non-experiential texts**	134	102	116	352
**Social reply texts**	85	66	85	236
**Information inquiry texts**	91	39	99	229
**Short texts with limited information**	174	100	164	438
**Interest-arousal texts**	2	0	3	5

## 4. Results

### 4.1 Characteristics of tourism image perception

#### 4.1.1. High-frequency word analysis: historical continuity, spatial environment, and everyday life jointly shape tourism image.

The top 20 high-frequency words indicate that visitors’ perceptions of the three sites primarily center on historical resources, the spatial environment, and everyday life experiences (Table 3). For Longwei Pass, high-frequency words such as “ancient city”, “history”, “historic site”, and “Nanzhao” point to memories of the historic pass and ancient city. Meanwhile, words such as “daily life”, “hustle and bustle”, “local people”, “coffee shop”, and “delicious” indicate that visitor perception has extended beyond viewing historical remains to include strolling through streets and alleys, everyday marketplace life, and light consumption experiences. The occurrence of “commercialization” also suggests that the involvement of tourism-related commercial formats may affect perceptions of authenticity. Therefore, the perceptual characteristics of Longwei Pass are mainly reflected in the image of a living neighborhood, continuously shaped by mountainous border-pass streets, and by alleys and everyday marketplace life.

**Table 3:** Top 20 high-frequency words of the three sites.

Rank	Longwei Pass	Frequency	Santai Mountain Historic Area	Frequency	Ming-Qing Tonghai Old Town	Frequency
**1**	Longwei Pass	408	Park	90	Xiushan Mountain	210
**2**	Ancient city	160	Mosque	49	Park	91
**3**	History	147	History	38	Confucian Temple	65
**4**	Historic site	139	Confucian Temple	38	History	62
**5**	Nanzhao	86	Dianchi Lake	32	Ancient city	57
**6**	Daily life	75	Bing Xin	31	Tasty	54
**7**	Hustle and bustle	56	Architecture	23	Qilu Lake	46
**8**	Coffee shop	55	Openness	23	Seagulls	39
**9**	Tasty	48	Environment	22	Culture	34
**10**	Erhai Lake	47	Hui people	19	Scenery	31
**11**	Street	46	Culture	18	County town	30
**12**	Architecture	45	Kuige Pavilion	17	Rice noodles	28
**13**	Cangshan Mountain	43	Worship	16	Mountain climbing	27
**14**	Commercialization	42	Religion	16	Local cuisine	26
**15**	Confucian Temple	40	Place	15	Good-looking	26
**16**	Gate tower	40	Distant view	13	Beautiful	26
**17**	Small shop	40	Period	13	Environment	24
**18**	Snacks	39	Daily life	11	Treasure	24
**19**	Local people	38	Good-looking	11	Daily life	23
**20**	Culture	38	Lin Huiyin	11	Architecture	23

For Santai Mountain Historic Area, the dominant high-frequency words include “park”, “mosque”, “Confucian Temple”, “Bing Xin”, and “Lin Huiyin”. These terms indicate that its image is jointly supported by mountainous public space, religious culture, and memories of notable figures. However, the distribution of high-frequency words is relatively dispersed, suggesting that its core tourism symbols are not yet prominent. Visitor perception depends more on the interplay among scattered cultural nodes and public recreational spaces. At the same time, words such as “daily life” and “environment” indicate that everyday life in surrounding communities remains an important context for visitor perception. Compared with Longwei Pass, Santai Mountain Historic Area has not yet developed a mature tourism industry or continuous commercial visitor routes. Its tourism image is mainly shaped by the juxtaposition of Santai Mountain Park, the surrounding community life, religious, cultural, and educational nodes, and public recreational activities.

For Ming-Qing Tonghai Old Town, words such as “Xiushan Mountain”, “park”, “Confucian Temple”, “ancient city”, and “Qilu” form a relatively stable image of a mountain–water ancient town. Words such as “delicious”, “rice noodles”, “local cuisine”, and “daily life” further strengthen the perception of local life and everyday consumption experiences. Compared with Longwei Pass and Santai Mountain Historic Area, the keywords for Ming-Qing Tonghai Old Town are more concentrated around its mountain–water ancient-town structure, Confucian Temple culture, and local life, indicating that its tourism image rests on a relatively strong and integrated resource base. Overall, visitor perceptions across the three sites reveal the interweaving of history and culture, spatial environment, and everyday life, while also showing differences in the organization of everyday life and perceptual structure.

#### 4.1.2. Semantic network analysis: hierarchical associations and case differences across space, setting, and meaning.

**Fig 4:**
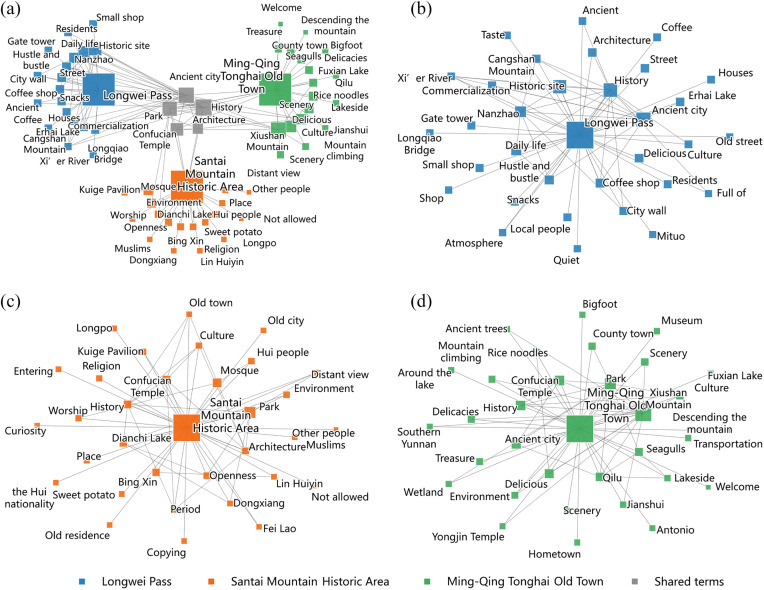
Semantic co-occurrence networks of high-frequency keywords.

Based on high-frequency word statistics, word co-occurrence networks were further constructed from visitor comments across the three sites to identify the associative structure of perceptual content. The integrated semantic network shows that the tourism image of the three sites is not formed by a single resource or isolated keywords. Instead, it is organized through multilayered associations among history and culture, the spatial environment, everyday life experiences, and emotional evaluations (Fig 4(a)). Words such as “ancient city”, “history”, “Confucian Temple”, “architecture”, and “park” constitute visitors’ basic spatial recognition of the neighborhoods. Words such as “snacks”, “coffee”, “mountain climbing”, “photography”, and “distant view” point to specific processes of recreation and consumption. Words such as “treasure”, “delicious”, “openness”, and “commercialization” reflect visitors’ subjective evaluations of spatial atmosphere, experience quality, and development status. These findings suggest that the tourism image of living HCNs does not remain at the level of resource recognition, but forms associations among spatial objects, activity settings, and local meanings.

The independent semantic networks across the three cases further reveal distinct patterns of perceptual organization. In the network of Longwei Pass, historical and cultural terms such as “ancient city”, “historic site”, “Nanzhao”, and “history” are closely associated with experiential terms related to everyday life, such as “hustle and bustle”, “daily life”, “local people”, “coffee shop”, and “delicious”. This indicates a strong continuity among the historic pass remains, the street-and-alley scale, and everyday marketplace life, through which visitors can move relatively easily from historical recognition to daily experience (Fig 4(b)). The semantic network of Santai Mountain Historic Area mainly revolves around words such as “park”, “mosque”, “Confucian Temple”, “Bing Xin”, “Kuige Pavilion”, and “worship”. This suggests that its tourism image is mainly supported by mountainous open space, religious sites, cultural and educational nodes, and surrounding community life, although the semantic associations among these resources remain relatively dispersed (Fig 4(c)). In Ming-Qing Tonghai Old Town, words such as “Xiushan Mountain”, “Confucian Temple”, “ancient city”, “Qilu”, and “scenery” form composite associations, indicating that its tourism image is built through the combined effects of the mountain–water structure, ancient-town historical continuity, and local life. At the same time, terms related to natural landscapes, ancient-town culture, and everyday life show a multi-centered juxtaposition, suggesting that visitor perception is more strongly characterized by the holistic recognition of composite resources than by a concentrated orientation toward a single theme (Fig 4(d)).

Overall, the semantic network results can be interpreted hierarchically through the HSSPR framework. The differences among the three types of cases are not merely differences in resource types; rather, they reflect differences in spatial form, the embeddedness of community life, and visitors’ perceptual pathways. These results provide a semantic basis for the subsequent sentiment analysis and IPA diagnosis.

#### 4.1.3. Sentiment analysis: sources of positive experience and deficiencies in service support.

Sentiment analysis was used to further identify visitors’ evaluative tendencies toward the tourism image of the three sites and potential deficiencies in it. Overall, comments on the three sites were dominated by positive sentiment: 1,866 comments were classified as positive, accounting for 73.46%; 438 were neutral, accounting for 17.24%; and 236 were negative, accounting for 9.29% (Table 4). Among the three sites, Longwei Pass had the highest proportion of positive comments, at 77.45%. Santai Mountain Historic Area recorded a positive sentiment rate of 67.72% and the highest proportion of neutral comments, suggesting that its valid experiential reviews contained more descriptive and weakly evaluative expressions. Ming-Qing Tonghai Old Town had a positive sentiment rate of 72.46%, but its proportion of negative comments was relatively high. Overall, the three sites all showed a solid foundation for visitor recognition, but differences in sentiment structure also indicate that the formation and consolidation of tourism image varied across cases.

**Table 4:** Statistical results of sentiment analysis.

Region	Number of valid comments	Positive	Percentage	Neutral	Percentage	Negative	Percentage
Longwei Pass	1020	790	77.45%	147	14.41%	83	8.14%
Santai Mountain Historic Area	536	363	67.72%	120	22.39%	53	9.89%
Ming-Qing Tonghai Old Town	984	713	72.46%	171	17.38%	100	10.16%
Total	2540	1866	73.46%	438	17.24%	236	9.29%

The high-frequency words associated with positive sentiment show that positive evaluations of the three sites were all related to historical and cultural atmosphere, local living settings, and spatial experience (Table 5), although their sources differed. Positive sentiment toward Longwei Pass depended on the continuous interaction between historic pass remains and everyday marketplace life. For Santai Mountain Historic Area, positive sentiment was mainly supported by religious, cultural, and educational nodes, mountainous public space, and the atmosphere of community life. Ming-Qing Tonghai Old Town was associated with the mountain–water ancient-town structure, Confucian Temple culture, and local dining experiences. These results suggest that positive sentiment across the three sites was not triggered by a single historical resource, but instead relied on different perceptual structures characterized by continuity, complexity, and integrity.

**Table 5:** Statistics of high-frequency words associated with positive sentiment.

Region	High-frequency words associated with positive sentiment	Main implications
**Longwei Pass**	Ancient city, history, historic site, Nanzhao, old street, hustle and bustle, daily life, coffee, small shop, delicious, quiet, rustic simplicity	Historical remains, street-and-alley life, light consumption experience
**Santai Mountain Historic Area**	Mosque, Confucian Temple, Dianchi Lake, Bing Xin, Kuige Pavilion, distant view, quiet, sunshine, culture, worship, history	Religious sites, historical memory, community public space
**Ming-Qing Tonghai Old Town**	Xiushan Mountain, Confucian Temple, ancient city, Qilu Lake, lakeside, seagulls, rice noodles, mountain climbing, scenery, local cuisine, temple	Mountain–city–lake landscape, educational and cultural traditions, local food

Negative evaluations were more often directed toward problems related to transport accessibility, public service facilities, signage and guidance, visitor route organization, and certain consumption experiences (Table 6). This indicates that visitor dissatisfaction was concentrated mainly in intermediary links such as spatial access, service capacity, and cultural interpretation.

**Table 6:** High-frequency words associated with negative sentiment and a summary of problems.

Region	High-frequency words associated with negative sentiment	Main problem types
**Longwei Pass**	Commercialization, shops, boring, houses, protection, parking, streetlights, toilets	Insufficient organization of commercial activities, inadequate supporting facilities, insufficient heritage interpretation
**Santai Mountain Historic Area**	Not allowed, mosque, toilets, old residence, boring, activities, openness, entering, unable to visit	Unclear access boundaries, inadequate public facilities, insufficient cultural interpretation
**Ming-Qing Tonghai Old Town**	Parking, transportation, crowded, congestion, lakeside, water quality, toilets, routes, far, tiring	Insufficient transport connections, discontinuous tour-route organization, inadequate lakeside services

### 4.2. Coding of tourism image perception elements and construction of the evaluation system

This study operationalized the progressive logic of the exploratory HSSPR framework (Space–Setting–Place–Realm) into a quantifiable analytical tool. Based on the semantic characteristics of UGC texts from the three sites and field investigation, an evaluation system was constructed, consisting of four perceptual dimensions and 16 specific perceptual elements (Table 7). The basic spatial support layer (A1–A4) represents the basic conditions required for visitors to enter the neighborhood and complete their visit. The recreational and consumption experience layer (B1–B4) captures living interactions and tourism consumption experiences within social settings. The historical landscape cognition layer (C1–C4) reflects visitors’ understanding of urban historic landscapes, local life, and culture. The emotional meaning and resonance layer (D1–D4) represents visitors’ value judgments, emotional identification, spiritual belonging, and behavioral intentions formed on the basis of their experiences. This system helps avoid the simplistic juxtaposition of heterogeneous elements such as space, commercial formats, culture, and emotion. Instead, following the hierarchical logic of HSSPR, it translates dispersed UGC expressions into a codable, comparable system of perceptual elements, thereby providing a basis for subsequent comparisons of perceptual differences and optimization priorities across the three sites.

**Table 7:** Coding system of tourism image perception elements based on the HSSPR framework.

HSSPR level	Perceived spatial dimension	Perceptual element	Keyword examples
**Space**	A Basic spatial support	A1 Location and transportation	Transportation, parking, public transport, self-driving, navigation, routes, distance, accessibility, location, address, etc.
		A2 Environmental sanitation	Sanitation, clean, tidy, garbage, dirty and disorderly, refreshing, unpleasant odor, cleanliness, etc.
		A3 Public service facilities	Toilets, restrooms, seats, lighting, streetlights, facilities, supporting facilities, maintenance, tourist center, etc.
		A4 Signage and tour guidance	Signboards, tour guidance, signage, entrance, exit, map, interpretation, information board, tour route, etc.
**Setting**	B Recreation and consumption experience	B1 Food and snacks	Snacks, delicious, rice noodles, barbecue, coffee, taste, delicacies, local cuisine, shop, owner, etc.
		B2 Photography and social media check-ins	Photography, photogenic spots, social media check-ins, photo spots, photography locations, easy-to-photograph scenes, Xiaohongshu, internet-famous sites, suitable for photography, etc.
		B3 Visiting order	Crowding, low crowding, tourists, queuing, crowded, bustling, quiet and peaceful, quiet, order, experiential quality, etc.
		B4 Commercial consumption	Commerce, commercialization, consumption, price, charges, admission tickets, expensive, cheap, free, value for money, etc.
**Place**	C Historical landscape cognition	C1 Traditional street-and-alley pattern	Old street, streets and alleys, alley, ancient city, ancient town, block, courtyard, vernacular dwellings, old houses, stone-paved road, etc.
		C2 Natural ecological landscape	Scenery, landscape, mountain, water, lake, lake view, nature, ecology, sunset, seagulls, sky, air, etc.
		C3 Historical and cultural relics	History, culture, historic site, historic buildings, temple, Confucian Temple, gate tower, Nanzhao, Tea Horse Ancient Road, etc.
		C4 Local folk life	Folk customs, folkways, the Bai nationality, local people, residents, everyday life atmosphere, hustle and bustle, market, marketplace, daily life, etc.
**Realm**	D Emotional meaning and resonance	D1 Physical and mental experience	Comfortable, relaxing, healing, pleasant and relaxing, leisurely, comfortable and peaceful, slow life, happy, quiet, fun, fatigue, etc.
		D2 Place identity	Distinctiveness, local flavor, authentic, cultural heritage, tradition, preservation, unique, locality, rustic simplicity, etc.
		D3 Memory and spiritual sustenance	Memory, recollection, nostalgia, homesickness, spirit, belief, mind, sustenance, inheritance, belonging, etc.
		D4 Revisit and recommendation intention	Recommendation, worthwhile, willingness to return, revisit intention, next time, intention to revisit, future opportunity, saving to favorites, interest arousal, planning to visit, etc.

On this basis, a five-level evaluation system was used to quantify the emotional tendencies expressed in visitor comments. According to the combined relationships among sentiment words, degree adverbs, and semantic contexts, the emotional intensity of each text was assigned a standardized score. After multiple rounds of review and consistency verification, the resulting scores provided data support for the IPA analysis (Table 8).

**Table 8:** Quantification criteria and scoring examples for perceptual elements.

Calculation method	Example comment text	Perceptual element	Assigned score
The positive probability is significantly high, and S is close to 5	The pickled-cabbage beef at a nearby restaurant was extremely delicious; the beef was very fresh and tender.	B1 Food and snacks	Approximately 5 points
The positive probability is relatively high, and S is usually 4–5	The old-town streets here are relatively well preserved.	C1 Traditional street-and-alley pattern	Approximately 4 points
The neutral probability is relatively high, and S is close to 3	A junction of the Tea Horse Ancient Road and the Southwest Silk Road.	A1 Location and transportation	Approximately 3 points
The negative probability increases, and S is usually 2–3	It takes a long time to walk uphill, which is rather tiring.	C2 Natural ecological landscape	Approximately 2 points
The negative probability is significantly high, and S is close to 1	Parking is very inconvenient, and there are no streetlights at night.	A3 Public service facilities	Approximately 1 point

### 4.3. Evaluation of tourism image perception and type differences

#### 4.3.1. Importance–performance scores.

Based on the IPA results, tourism image perception across the three sites showed shared strengths, common deficiencies, and type-specific differences among the HSSPR-coded perceptual elements (Table 9). In terms of shared strengths, C3 (historical and cultural relics) was located in Quadrant I (high attention–high performance) in all three cases, indicating that this perceptual element remains a core basis for positive evaluations of living HCNs. In terms of common deficiencies, B1 (food and snacks) was located in Quadrant IV (high attention–low performance) in all three cases. This suggests that although local food is an important way for visitors to engage with the everyday life of the neighborhoods, the existing supply has not yet been consistently leveraged as an experiential advantage.

**Table 9:** IPA evaluation results for tourism image perceptual elements.

Perceptual dimension	Element code	Longwei Pass				Santai Mountain Historic Area				Ming-Qing Tonghai Old Town			
		frequency (n)	Importance (I)	Performance (P)	IPA quadrant	frequency (n)	Importance (I)	Performance (P)	IPA quadrant	frequency (n)	Importance (I)	Performance (P)	IPA quadrant
A	A1	240	9.4637	4.2256	Ⅳ	90	11.0429	4.1380	Ⅰ	172	9.6684	4.0318	Ⅳ
	A2	10	0.3943	4.1796	Ⅲ	9	1.1043	3.9331	Ⅲ	10	0.5621	4.4756	Ⅱ
	A3	20	0.7886	4.3641	Ⅱ	29	3.5583	4.0026	Ⅲ	25	1.4053	4.4520	Ⅱ
	A4	39	1.5379	4.2894	Ⅲ	17	2.0859	4.0676	Ⅱ	18	1.0118	3.9439	Ⅲ
B	B1	296	11.6719	4.3034	Ⅳ	59	7.2393	3.8351	Ⅳ	192	10.7926	3.9867	Ⅳ
	B2	142	5.5994	4.3639	Ⅱ	52	6.3804	3.8538	Ⅳ	84	4.7218	4.0858	Ⅲ
	B3	145	5.7177	4.5010	Ⅱ	46	5.6442	4.1609	Ⅱ	65	3.6537	4.4502	Ⅱ
	B4	180	7.0978	4.1208	Ⅳ	47	5.7669	3.8438	Ⅲ	116	6.5205	3.8527	Ⅳ
C	C1	244	9.6215	4.3715	Ⅰ	23	2.8221	3.8737	Ⅲ	68	3.8224	4.0106	Ⅲ
	C2	361	14.2350	4.2446	Ⅳ	189	23.1902	4.0847	Ⅰ	491	27.5998	4.1052	Ⅳ
	C3	233	9.1877	4.4358	Ⅰ	70	8.5890	4.2502	Ⅰ	153	8.6003	4.3577	Ⅰ
	C4	166	6.5457	4.4139	Ⅰ	31	3.8037	4.0239	Ⅲ	52	2.9230	4.0528	Ⅲ
D	D1	211	8.3202	4.4492	Ⅰ	60	7.3620	4.2640	Ⅰ	136	7.6447	4.1587	Ⅳ
	D2	86	3.3912	4.6584	Ⅱ	24	2.9448	4.2105	Ⅱ	49	2.7544	4.4943	Ⅱ
	D3	29	1.1435	4.2223	Ⅲ	35	4.2945	4.0179	Ⅲ	15	0.8432	4.2849	Ⅱ
	D4	134	5.2839	4.4431	Ⅱ	34	4.1718	4.3360	Ⅱ	133	7.4761	4.4804	Ⅰ

Note: The perceptual dimensions and element codes are defined in Table 7. Frequency (n) indicates the frequency of element mentions; IPA quadrants are defined as follows: Ⅰ = maintain advantages; Ⅱ = moderate maintenance; Ⅲ = low priority; Ⅳ = priority improvement.

The differences were mainly reflected in Space-layer access conditions, Place-layer natural and ecological resources, and Setting- and Realm-related elements of everyday-life experience. In Longwei Pass and Ming-Qing Tonghai Old Town, C2 (natural ecological landscape) and A1 (location and transportation) were both located in Quadrant IV, reflecting a high-attention–low-performance mismatch among mountain–water resources, neighborhood accessibility, and the organization of actual visits. In Longwei Pass, C1 (traditional street-and-alley pattern), C4 (local folk life), and D1 (physical and mental experience) were all located in Quadrant I, indicating the perceptual advantage generated by the continuity of street-and-alley life. In Santai Mountain Historic Area, A1 (location and transportation), C2 (natural ecological landscape), C3 (historical and cultural relics), and D1 (physical and mental experience) were all located in Quadrant I, whereas B1 (food and snacks) and B2 (photography and social media check-ins) were located in Quadrant IV. This indicates that its public recreational spaces and historical, cultural, and educational nodes have basic appeal, while its consumption settings and social-sharing potential are not yet fully translated into perceptual advantages.

Overall, the perceptual differences among the three sites were to some extent associated with the degree of tourism development, namely Ming-Qing Tonghai Old Town > Longwei Pass > Santai Mountain Historic Area. However, this relationship does not follow a simple linear pattern; rather, it is jointly shaped by the organization of spatial resources, the embedding of everyday-life settings, the formation of place-based understanding, and the capacity to translate services into the visitor experience.

#### 4.3.2. Differences in the perceptual structure of tourism image and their formation logic.

**Fig 5:**
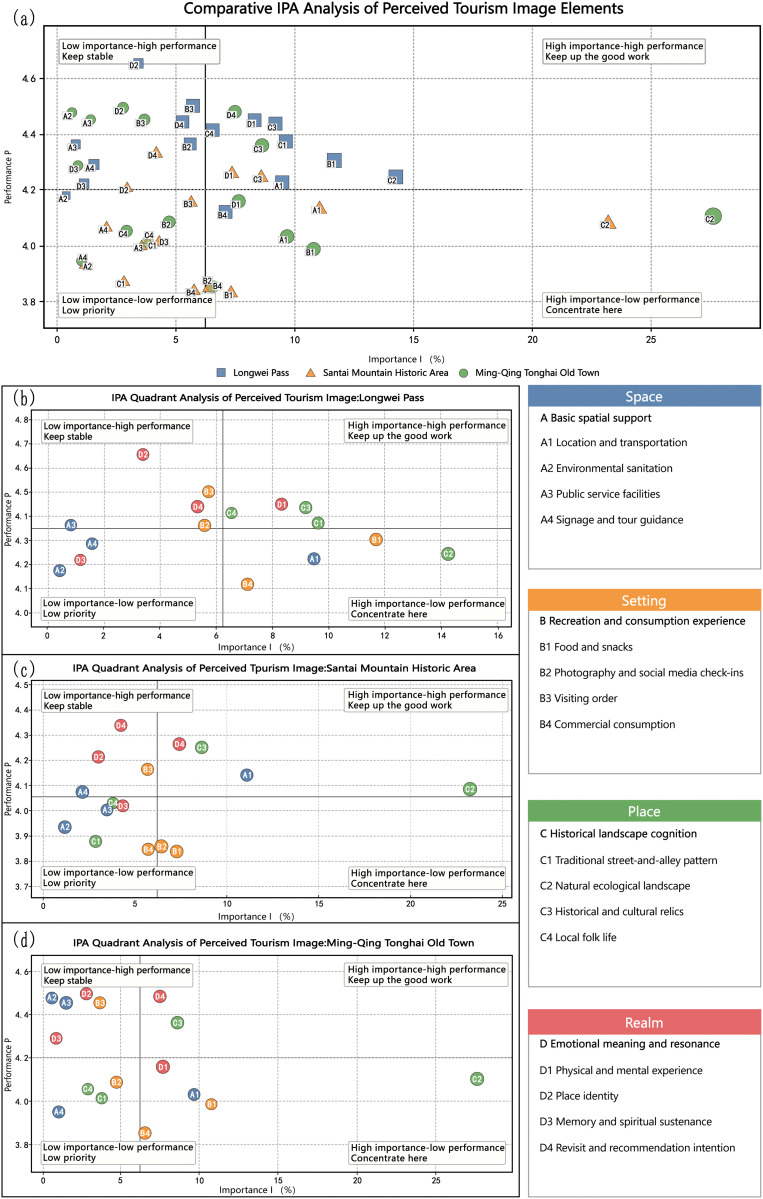
IPA quadrant analysis of tourism image perceptual elements.

Based on the quadrant classification above, Fig 5(a) presents the overall distribution of HSSPR-coded perceptual elements across the three sites in the coordinate system of “attention salience–experiential performance”. The results show that high-attention elements within different HSSPR layers do not necessarily correspond to high experiential performance. This indicates that the tourism image of living HCNs depends not only on resource endowment or tourism development level, but also on how Space-layer access conditions, Setting-layer activities, and Place-layer resources are organized into accessible, understandable, and experienceable systems. The following analysis further explains the perceptual differences among the three cases based on their IPA distributions.

The IPA results for Longwei Pass show a strong linkage among Place recognition, Setting participation, and Realm-level experience (Fig 5(b)). C1 (traditional street-and-alley pattern), C3 (historical and cultural relics), C4 (local folk life), and D1 (physical and mental experience) were all located in Quadrant I, indicating a stable perceptual association among historic pass remains, traditional streets and alleys, and the everyday marketplace life of old Xiaguan. Visitors can therefore move relatively easily from historical recognition to street-and-alley strolling and everyday-life experience. However, C2 (natural ecological landscape), B1 (food and snacks), B4 (commercial consumption), and A1 (location and transportation) remained in the priority improvement quadrant, suggesting that the Cangshan–Erhai setting, light-consumption formats, and neighborhood accessibility have not yet been fully transformed into stable experiential advantages. The key issue for Longwei Pass is therefore to improve transport access, heritage interpretation, and light-consumption organization while maintaining the authenticity of street-and-alley life.

The perceptual structure of the Santai Mountain Historic Area is more complex, with advantages concentrated in Space-, Place-, and Realm-layer elements but weaknesses remaining in Setting-layer consumption and social-sharing elements (Fig 5(c)). A1 (location and transportation), C2 (natural ecological landscape), C3 (historical and cultural relics), and D1 (physical and mental experience) were all located in Quadrant I, indicating that its mountainous environment and religious, cultural, and educational nodes jointly constitute its basic appeal. However, B1 (food and snacks) and B2 (photography and social media check-ins) fell into the priority improvement quadrant, while B4 (commercial consumption), C1 (traditional street-and-alley pattern), and D3 (memory and spiritual sustenance) were mainly located in the low-priority quadrant. D2 (place identity) showed relatively high performance but limited attention. These results suggest that although visitors can recognize its natural environment and historical nodes, they have difficulty forming continuous consumption experiences, social-media-based memories, and an integrated understanding of place. This pattern is related to its limited tourism development, the point-like distribution of resources, the relatively clear access boundaries of religious, cultural, and educational spaces, and the fact that community life has not yet been effectively organized into tourism narratives.

Ming-Qing Tonghai Old Town has a relatively strong Place-layer resource foundation, but its transformation into continuous Setting- and Realm-level experiences remains insufficient (Fig 5(d)). C3 (historical and cultural relics) and D4 (revisit and recommendation intention) were located in Quadrant I, suggesting that Confucian Temple culture, the ancient-town pattern, and local life are highly recognizable and can stimulate a certain level of recommendation intention. However, C2 (natural ecological landscape), as the perceptual element with the highest attention salience, did not fully meet visitor expectations regarding experiential performance. A1 (location and transportation), B1 (food and snacks), and B4 (commercial consumption) were also located in the priority improvement quadrant, revealing deficiencies in access, consumption, and service support. C1 (traditional street-and-alley pattern) and C4 (local folk life) had not yet developed prominent experiential advantages. Although Xiushan Mountain, the old town, and Qilu Lake form a relatively complete mountain–water, cultural, and educational structure, visitor-route connections, transport transfers, and service capacity still limit the transformation of the overall resource system into a continuous visitor experience.

## 5. Discussion

### 5.1. Explanatory support of the HSSPR framework for the perceptual hierarchy

The hierarchical mapping of UGC texts from the three sites supports the progressive pattern of visitor perception in living HCNs. Visitor perception is not determined solely by the appeal of historical resources or the provision of commercial services; rather, it is gradually formed through spatial access, setting participation, place understanding, and meaning expression. The physical accessibility and infrastructure of the Space layer constitute the material foundation of the overall perceptual map. As visitors become more deeply involved in the everyday marketplace life and recreational consumption of the Setting layer, multisensory embodied experiences are activated, further promoting their subjective understanding of urban historic landscapes and cultural authenticity at the Place layer. Through emotional accumulation, this understanding is eventually transformed at the Realm layer into deeper spiritual resonance and intentions to revisit. These findings empirically address part of the explanatory gap in the classic psychological destination image model proposed by Baloglu et al., particularly regarding its application to three-dimensional physical space and everyday social contexts, and show that the organizational efficiency of underlying material space and living settings can directly constrain or facilitate the transformation of higher-order cultural and spiritual images.

### 5.2. The mismatch between attention salience and experiential performance highlights the importance of intermediary links

The adaptive IPA-based joint diagnosis using mention frequency as attention salience and TextCNN sentiment scores as experiential performance revealed a shared structural mismatch in visitor perception across the three sites. Historical and cultural relics, physical and mental experience, and revisit and recommendation intention generally performed well, indicating that historical resources and local atmosphere remain important foundations for the formation of a positive tourism image in living HCNs. By contrast, food and snacks showed a high-attention–low-performance mismatch across all three sites, whereas location and transportation, commercial consumption, natural ecological landscape, and physical and mental experience showed the same pattern in some cases. This result suggests that the main bottlenecks hindering tourism image enhancement do not stem primarily from a lack of core historical resources, but are concentrated in intermediary links such as spatial access, service capacity, visitor-route organization, and cultural interpretation. In other words, whether historical resources can be successfully translated into stable positive experiences hinges on visitors’ ability to smoothly enter, stay in, understand, and participate in neighborhood life.

### 5.3. Case differences indicate the need for differentiated experience organization in similar neighborhoods

The comparison of the three cases shows that living HCNs are not homogeneous tourism spaces, and their image formation cannot rely solely on a uniform logic of resource display or attraction-based development. Longwei Pass, Santai Mountain Historic Area, and Ming-Qing Tonghai Old Town present perceptual structures characterized by continuity, complexity, and integrity, respectively, indicating that spatial form, the organization of community life, and the degree of tourism development jointly shape visitors’ perceptual pathways and experiential evaluations. These differences provide direct empirical evidence for fine-grained spatial governance: Longwei Pass should maintain a balance among historic pass remains, street-and-alley life, and moderate consumption while avoiding the erosion of everyday-life authenticity through excessive commercialization; Santai Mountain Historic Area should strengthen visitor-route organization and narrative linkage among the park, community life, and religious, cultural, and educational nodes; and Ming-Qing Tonghai Old Town should improve visitor-route connections and service capacity among the mountain, town, and lake so that its overall resource advantages can be translated into a continuous visitor experience. Overall, image formation in living HCNs should shift from simple resource display to the organization of experience, while improving the conditions through which visitors enter, understand, and experience local culture on the basis of maintaining the continuity of everyday life.

## 6. Conclusions and prospects

Taking Longwei Pass in Dali, Santai Mountain Historic Area in Chenggong, Kunming, and Ming-Qing Tonghai Old Town in Tonghai as empirical cases, this study examined the structural characteristics, type-specific differences, and optimization bottlenecks of tourism image perception in living HCNs by integrating UGC text analysis, TextCNN-based sentiment recognition, and adaptive IPA analysis. The main conclusions are as follows.

1. The HSSPR framework proposed in this study can translate dispersed visitor perception expressions in living HCNs into a progressive and hierarchical evaluation system. Based on the four layers of Space, Setting, Place, and Realm, this study constructed four dimensions—basic spatial support, recreational and consumption experience, historical landscape cognition, and emotional meaning and resonance—together with 16 perceptual elements. This system shows that tourism image perception in living HCNs is not a single-attribute evaluation, but a composite process jointly shaped by spatial access, setting participation, cultural understanding, and meaning expression.
2. The three cases all show the shared characteristics of historical continuity, spatial environment, and everyday life being interwoven, but their perceptual structures differ clearly by type. The tourism image of Longwei Pass is mainly reflected in the continuous formation of historic pass remains, traditional streets and alleys, and everyday marketplace life. Santai Mountain Historic Area is characterized by the composite interaction among its mountainous environment, religious, cultural, and educational nodes, public recreation, and community life. Ming-Qing Tonghai Old Town reflects the integrated coupling of its mountain–water structure, ancient-town historical continuity, and local life. These findings indicate that differences in the tourism image of living HCNs are jointly influenced by spatial form, the organization of local life, and the degree of tourism development.
3. The adaptive IPA results show that historical and cultural relics, physical and mental experience, and revisit and recommendation intention are perceptual elements that are more likely to generate positive evaluations across the three sites. However, high-attention–low-performance mismatches are concentrated mainly in elements of basic spatial support and recreational and consumption experience. This suggests that the bottlenecks hindering the enhancement of the tourism image of living HCNs do not primarily lie in a lack of historical resources. Instead, they are concentrated in intermediary links such as spatial access, service capacity, visitor-route organization, and cultural interpretation.

This study has three limitations. First, the data were collected from public online platforms and therefore reflect visitor perceptions expressed in online contexts. They may be affected by user composition and platform posting mechanisms and cannot fully represent all visitors. Second, this study used element frequency and TextCNN sentiment scores to represent attention salience and experiential performance, respectively. These are proxy measurements based on UGC and are not strictly equivalent to traditional questionnaires. Third, as an exploratory tool, the applicability and explanatory boundaries of the HSSPR framework still need to be tested across different neighborhood types, conservation levels, and stages of tourism development. Future research could combine questionnaires, interviews, field observations, visitor trajectories, and other multi-source data to cross-validate perceptual processes and spatial triggering mechanisms, thereby improving the framework's applicability and the robustness of its measurements.

## Supporting information

S1 File De-identified supporting data and reproducible analysis materials. The ZIP archive contains de-identified and English-translated review-level data for the three study sites; preprocessing resources and outputs; TextCNN training, validation, and test data, model weights, evaluation records, and Python scripts; sentiment-recognition results; IPA calculation materials; semantic-network analysis files; workflow documentation; and a file manifest.
(ZIP)
