# Supplementary material for: Tourism image perception of living historical and cultural neighborhoods under the HSSPR framework: A comparative study of three cases in Yunnan, China
Source: PLoS One. 2026 Aug 14;21(8):e0355218. doi: 10.1371/journal.pone.0355218 (PMC13475901; doi:10.1371/journal.pone.0355218)
Supplement: Supplementary file 1 [file pone.0355218.s001.zip › Supporting Information/01_Documentation/HSSPR_UGC_TextCNN_IPA_Workflow_Guide_EN.docx]

**Complete Workflow Guide for UGC Text Processing, Sentiment Recognition, and IPA Analysis under the HSSPR Framework**

English version of the adjusted workflow documentation

# 1. Purpose of This Integrated Guide

This guide reorganizes the existing technical route documents into a complete and reproducible workflow aligned with the research logic of the main article. The workflow is arranged by data source, text processing, semantic identification, sentiment computation, perceptual element coding, IPA diagnosis, and result interpretation.

# 2. Research Logic and Overall Workflow

The study interprets the tourism image of living historic cultural neighborhoods through the progressive HSSPR framework. User-generated comments are used as observational materials of tourist perception. The analysis first identifies high-frequency terms and semantic co-occurrence, then derives experiential performance through sentiment recognition, and finally integrates vocabulary, sentiment, and perceptual elements into IPA quadrants.

| **Step** | **Stage** | **Tools or Files** | **Main Outputs** |
| --- | --- | --- | --- |
| 1 | De-identified source review aggregation | De-identified and English-translated review-level data derived from Dianping, Xiaohongshu, and Ctrip | Traceable three-site review-level supporting dataset |
| 2 | Text preprocessing | Preprocessing script; stopwords.txt; user_dict.txt | Cleaned reviews, tokenized results, corpus files, and high-frequency word statistics |
| 3 | Core term screening and classification | Three-site core term screening and four-dimensional classification workbook | HSSPR four-level and 16-element perceptual coding system |
| 4 | Rule-based labeling and training set preparation | Sentiment lexicon and scoring rules; sample preparation script | Manually checked labeled samples and train/validation/test splits |
| 5 | TextCNN sentiment recognition | Model training and prediction scripts | Model weights, test report, and CNN sentiment recognition results |
| 6 | Interactive reply filtering | Filtering by sample type, including replies, Q&A, and weak-information texts | Main sentiment sample for IPA analysis |
| 7 | IPA computation | IPA analysis script and 16-element IPA workbook | Importance I, performance P, and quadrant classification for 16 elements |
| 8 | Visualization and interpretation | Redrawn IPA figures, semantic network figures, and statistical summaries | Evidence for the results, discussion, and development-stage diagnosis |

# 3. Data Directory and File Organization

| Directory | Contents | Purpose |
| --- | --- | --- |
| 00_Deidentified_Source_Review_Data | De-identified and English-translated review-level source data for the three study sites, plus a short data note | Source data transparency and reproducibility without direct user identifiers |
| 01_Documentation | English workflow guide | Documentation for interpretation, replication, and handoff |
| 02_Preprocessed_Datasets | Stopwords, user dictionary, preprocessing outputs, tokenized corpora, high-frequency word tables, and the core-term classification master table | Text cleaning, segmentation, high-frequency word statistics, and core-term screening |
| 03_TextCNN_Workflow | Training labels, train/validation/test files, label mapping, vocabulary, model weights, evaluation records, and numbered Python scripts | Rule labeling, sample splitting, model training, model evaluation, and batch prediction |
| 04_CNN_Sentiment_Recognition_Results | Site-level and three-site CNN sentiment-recognition results after filtering interactive replies | Inputs for performance calculation and sentiment-structure interpretation |
| 05_IPA_16_Element_Analysis_Results | English IPA calculation workbook and publication-quality IPA figures | Importance-performance calculation and comparative quadrant diagnosis |
| 06_Semantic_Network_Analysis | Keyword-selection tables, scripts, final network figures, and node/edge metric workbooks | Semantic co-occurrence analysis and publication-quality visualization |

# 4. Step 1: Raw Data Aggregation and Text Preprocessing

The study draws on reviews collected from Dianping, Xiaohongshu, and Ctrip. For journal submission and reproducibility, this package provides de-identified and English-translated review-level source data rather than the original unprocessed crawler exports. The preprocessing stage standardizes fields by site, including site, platform, place name, review hierarchy, posting time, rating, source file, original review, cleaned review, and tokenized result. Stopwords are removed, while custom feature terms preserve place names, historical-cultural terms, recreation and consumption terms, and local-life expressions.

| **Site** | **Preprocessed Reviews** | **Result File** | **Top High-Frequency Terms** |
| --- | --- | --- | --- |
| Longwei Pass | 1506 | 02_Preprocessed_Reviews_Longwei_Pass.xlsx | Longwei Pass, ancient city, history, relic site, Nanzhao, daily life, hustle and bustle, coffee shop, delicious, Erhai Lake |
| Ming-Qing Tonghai Old Town | 1451 | 02_Preprocessed_Reviews_Tonghai.xlsx | Xiushan Mountain, park, Confucian Temple, history, ancient city, delicious, Qilu, seagulls, culture, scenery |
| Santai Mountain Historic Area | 843 | 02_Preprocessed_Reviews_Santai_Mountain.xlsx | Park, mosque, not allowed, Confucian Temple, history, Dianchi Lake, Bing Xin, openness, architecture, environment |

# 5. Step 2: Core Terms and the HSSPR Perceptual Element System

Core terms are selected from high-frequency word statistics to represent spatial recognition, recreation experience, historical-cultural cognition, and emotional meaning. The classified terms transform unstructured review text into perceptual elements under the HSSPR framework and connect semantic networks, sentiment-based performance, and IPA diagnosis.

| **Worksheet** | **Records** | **Description** |
| --- | --- | --- |
| Full table with deleted terms | 360 | All candidate terms and deletion/retention judgment |
| Core terms | 294 | Terms retained for the core-term screening process |
| Classified core terms | 134 | Core terms mapped to HSSPR dimensions or specific perceptual elements |
| Terms for manual review | 160 | Terms requiring contextual confirmation |
| Four-dimensional comparison | 3 | Comparison of the three sites across four dimensions |
| Longwei / Santai / Tonghai sheets | 104 / 98 / 92 | Site-level core term screening and classification results |

| **HSSPR Level** | **Perceived Spatial Dimension** | **Code** | **Perceptual Element** |
| --- | --- | --- | --- |
| Space | A Basic spatial support | A1 | Location and transportation |
| Space | A Basic spatial support | A2 | Environmental sanitation |
| Space | A Basic spatial support | A3 | Public service facilities |
| Space | A Basic spatial support | A4 | Signage and tour guidance |
| Setting | B Recreation and consumption experience | B1 | Food and snacks |
| Setting | B Recreation and consumption experience | B2 | Photography and social media check-ins |
| Setting | B Recreation and consumption experience | B3 | Visiting order |
| Setting | B Recreation and consumption experience | B4 | Commercial consumption |
| Place | C Historical landscape cognition | C1 | Traditional street-and-alley pattern |
| Place | C Historical landscape cognition | C2 | Natural ecological landscape |
| Place | C Historical landscape cognition | C3 | Historical and cultural relics |
| Place | C Historical landscape cognition | C4 | Local folk life |
| Realm | D Emotional meaning and resonance | D1 | Physical and mental experience |
| Realm | D Emotional meaning and resonance | D2 | Place identity |
| Realm | D Emotional meaning and resonance | D3 | Memory and spiritual sustenance |
| Realm | D Emotional meaning and resonance | D4 | Revisit and recommendation intention |

# 6. Step 3: Sentiment Lexicon, Rule-Based Labels, and Training Samples

Sentiment analysis does not directly use star ratings as performance values. Instead, weak labels are first generated from the sentiment lexicon and scoring rules, then manually checked and used to train the TextCNN model. The rules distinguish positive, neutral, and negative expressions and account for negation, degree adverbs, contrastive structures, and contextual terms.

| **Rule Type** | **Recognized Content** | **Function in the Workflow** |
| --- | --- | --- |
| Positive | Positive experiential expressions such as delicious, beautiful, worthwhile, comfortable, convenient, distinctive | Indicates higher perceived performance |
| Neutral | Objective description, place information, route information, or text without an obvious attitude | Used as an intermediate performance category |
| Negative | Negative expressions such as inconvenient, disappointing, not allowed, crowded, dirty, or over-commercialized | Indicates lower perceived performance |
| Adjustment rules | Negation words, degree adverbs, contrast markers, consecutive emotion words, and contextual terms | Adjusts the direction or strength of the initial sentiment |
| Filtering rules | Interactive replies, Q&A, weak-information short texts, and recommendation-intention-only texts | Prevents non-evaluative content from diluting performance scores |

| **Dataset** | **Samples** | **Label Structure** |
| --- | --- | --- |
| All manually labeled samples | 7532 | Positive 4035; Neutral 2766; Negative 731 |
| Training set | 6025 | Positive 3228; Neutral 2212; Negative 585 |
| Validation set | 753 | Positive 403; Neutral 277; Negative 73 |
| Test set | 754 | Positive 404; Neutral 277; Negative 73 |

# 7. Step 4: TextCNN Training and Batch Sentiment Recognition

TextCNN classifies cleaned and tokenized review texts into positive, neutral, or negative sentiment. The workflow retains the vocabulary, label mapping, training records, model weights, test report, and confusion matrix, making the path from training samples to prediction results traceable.

| Stage | Script | Output or Use |
| --- | --- | --- |
| Sample preparation | 01_sample_preparation.py | Cleaned manually labeled samples and train/validation/test splits |
| Model training | 02_model_training.py | Vocabulary, label mapping, training records, model weights, and test report |
| Site-specific prediction | 03_new_review_prediction.py | Sentiment prediction for a specified site |
| Full prediction | 04_cnn_full_prediction.py | Three-site sentiment prediction under the full-data scope |
| Prediction after interaction filtering | 05_cnn_prediction_after_xiaohongshu_filtering.py | Main sentiment sample for IPA analysis |
| IPA linkage | 06_ipa_analysis.py | Matching sentiment results with 16 elements and calculating I and P values |

| **Metric** | **Value** |
| --- | --- |
| Loss | 0.3768 |
| Accuracy | 0.8634 |
| Macro Precision | 0.8026 |
| Macro Recall | 0.7828 |
| Macro F1 | 0.7918 |

# 8. Step 5: Interactive Reply Filtering and Main Sentiment Results

Because platforms such as Xiaohongshu contain many replies, Q&A texts, and weak-information posts, these texts are closer to social interaction than on-site tourism experience evaluation. The performance calculation therefore uses the main sentiment sample after removing interactive replies.

| **Site** | **Positive N** | **Positive Share** | **Neutral N** | **Neutral Share** | **Negative N** | **Negative Share** |
| --- | --- | --- | --- | --- | --- | --- |
| Longwei Pass | 790 | 77.45% | 147 | 14.41% | 83 | 8.14% |
| Tonghai | 713 | 72.46% | 171 | 17.38% | 100 | 10.16% |
| Santai Mountain | 363 | 67.72% | 120 | 22.39% | 53 | 9.89% |

# 9. Step 6: IPA Calculation and Quadrant Diagnosis

IPA converts the frequency with which tourists mention a perceptual element into importance I and converts the sentiment score of related reviews into performance P. The 16 HSSPR perceptual elements are used as IPA units for each site and for the three-site comparison.

| **Item** | **Formula or Rule** | **Meaning** |
| --- | --- | --- |
| Single-review sentiment score | S_i = 5 x P(positive) + 3 x P(neutral) + 1 x P(negative) | Maps CNN prediction probabilities to a 1-5 performance score |
| Element frequency | F_n = sum M_in | M_in indicates whether review i matches element n |
| Importance | I_n = F_n / sum F_n x 100% | Relative attention of an element among all element mentions |
| Performance | P_n = sum(M_in x S_i) / F_n | Average sentiment performance of reviews matching the element |
| Quadrant division | The mean I and mean P within each site are used as split lines | Forms four categories: keep up, concentrate here, keep stable, and low priority |

| **Site** | **Matched Element Frequency** | **Mean I** | **Mean P** | **Top 5 Importance Elements** |
| --- | --- | --- | --- | --- |
| Longwei Pass | 2536 | 6.25 | 4.3492 | C2 Natural ecological landscape 14.235%; B1 Food and snacks 11.6719%; C1 Traditional street-and-alley pattern 9.6215%; A1 Location and transportation 9.4637%; C3 Historical and cultural relics 9.1877% |
| Tonghai | 1779 | 6.25 | 4.2015 | C2 Natural ecological landscape 27.5998%; B1 Food and snacks 10.7926%; A1 Location and transportation 9.6684%; C3 Historical and cultural relics 8.6003%; D1 Physical and mental experience 7.6447% |
| Santai Mountain | 815 | 6.25 | 4.0560 | C2 Natural ecological landscape 23.1902%; A1 Location and transportation 11.0429%; C3 Historical and cultural relics 8.5890%; D1 Physical and mental experience 7.3620%; B1 Food and snacks 7.2393% |

| **Site** | **Quadrant Structure** | **Interpretive Position** |
| --- | --- | --- |
| Longwei Pass | Keep stable 5; Concentrate here 4; Keep up 4; Low priority 3 | Medium-high development, historic-district activation type. Historical culture, street pattern, and local-life experience have formed a stable image, while transportation, food consumption, and ecological experience still require optimization. |
| Tonghai | Concentrate here 5; Keep stable 5; Low priority 4; Keep up 2 | Medium development, resource-driven type. Mountain-city-lake resources are attractive, but transport connection, food consumption, route organization, and experiential conversion lag behind resource appeal. |
| Santai Mountain | Low priority 6; Keep up 4; Keep stable 4; Concentrate here 2 | Low-to-medium development, cultural-resource potential cultivation type. Natural, historical, religious, and public leisure values are partly perceived, but consumption scenes, communication nodes, and recreational activities remain weak. |

# 10. Outputs and Linkage to the Paper

| Output Type | Result File | Use in the Paper |
| --- | --- | --- |
| High-frequency terms and semantic networks | 04_High_Frequency_Words_*.xlsx; files under 06_Semantic_Network_Analysis | Sections 4.1.1 and 4.1.2: tourist perception content and co-occurrence structure |
| Core-term classification system | 03_Three_Site_Core_Term_Classification_Master_Table.xlsx | Section 4.2: construction of the HSSPR four-dimensional and 16-element system |
| Sentiment recognition | Three_Sites_CNN_Sentiment_Statistics_After_Interactive_Reply_Filtering.xlsx | Section 4.1.3: overall sentiment tendency and performance basis |
| IPA analysis | Three_Sites_IPA_16_Element_Calculation_Results_20260528_201741.xlsx and the English IPA PNG/PDF figures | Section 4.3: importance, performance, and quadrant diagnosis |
| Figures | English semantic-network and IPA figures, including SCI_reference_style_2x2_preview_2EN_compact.png | Publication-quality English figures supporting the manuscript results |

# Appendix A: Key File List

| Category | Path | Description |
| --- | --- | --- |
| De-identified source review data | 00_Deidentified_Source_Review_Data | English-translated review-level supporting data for the three study sites; direct user identifiers and platform links are excluded |
| Preprocessing script | 02_Preprocessed_Datasets\preprocessing_script.py | Field standardization, cleaning, tokenization, corpus generation, and high-frequency term output |
| Stopwords | 02_Preprocessed_Datasets\01_Stopword_Data\stopwords.txt | Filters common terms without analytical meaning |
| User dictionary | 02_Preprocessed_Datasets\02_User_Dictionary\user_dict.txt | Preserves place names, historical-cultural terms, and scenario-specific terms |
| Core-term classification | 02_Preprocessed_Datasets\04_Core_Term_Classification_Results\03_Three_Site_Core_Term_Classification_Master_Table.xlsx | Basis for HSSPR element coding |
| TextCNN process files | 03_TextCNN_Workflow\00_TextCNN_Process_Files | Training, validation, and test sets; label mapping; vocabulary; weights; records; report; and confusion matrix |
| Sentiment results | 04_CNN_Sentiment_Recognition_Results | Site-level and three-site summary sentiment-recognition results |
| IPA results | 05_IPA_16_Element_Analysis_Results | English IPA calculation workbook and publication-quality figures |
| Semantic-network results | 06_Semantic_Network_Analysis | Keyword-selection tables, scripts, final figures, and node/edge metrics |
