# Supplementary material for: Tourism image perception of living historical and cultural neighborhoods under the HSSPR framework: A comparative study of three cases in Yunnan, China
Source: PLoS One. 2026 Aug 14;21(8):e0355218. doi: 10.1371/journal.pone.0355218 (PMC13475901; doi:10.1371/journal.pone.0355218)
Supplement: Supplementary file 1 [file pone.0355218.s001.zip › Supporting Information/05_IPA_16_Element_Analysis_Results/IPA_redrawn_English_SCI_compact/Longwei_Pass_IPA_EN_SCI_compact.pdf]

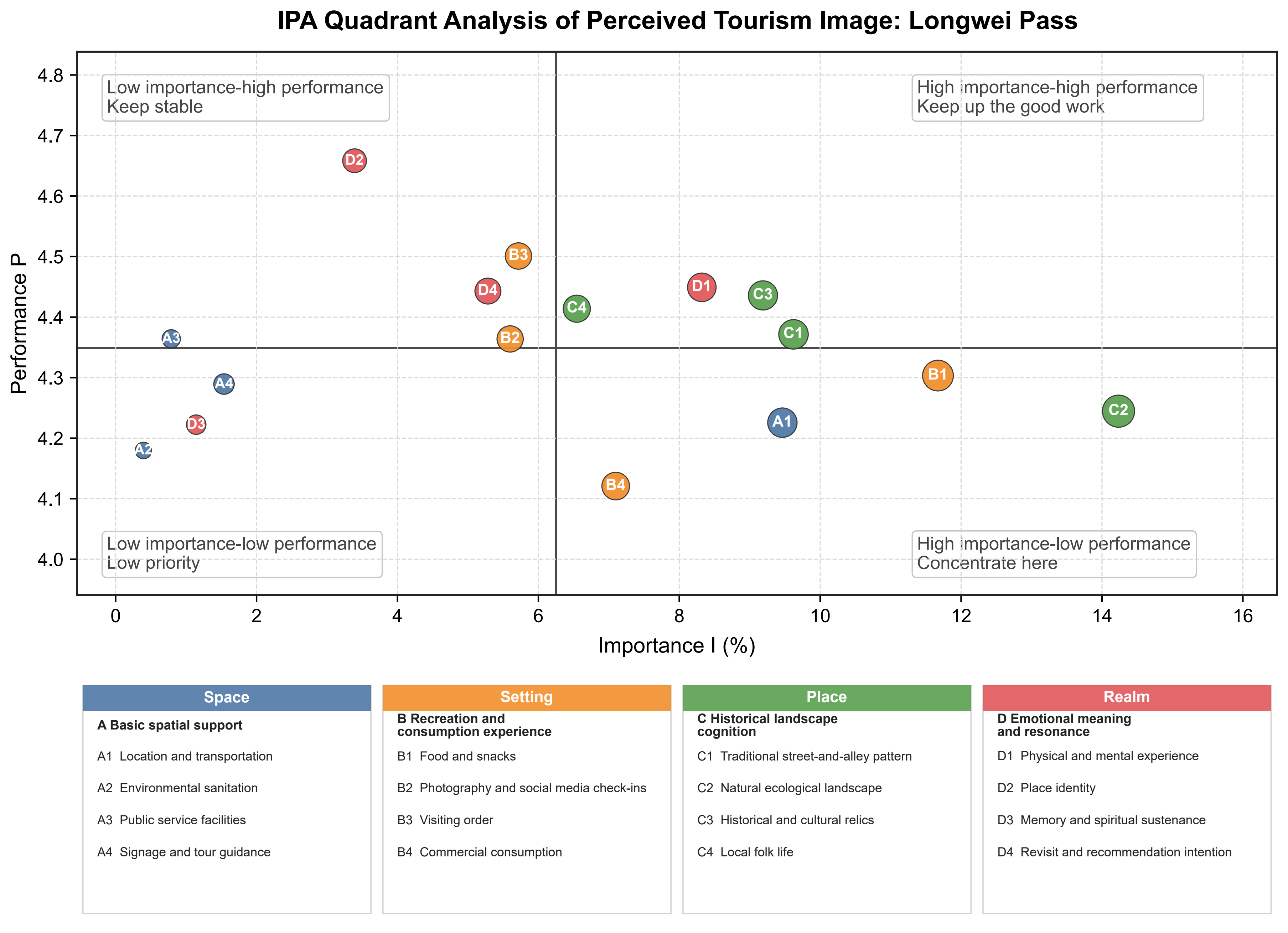
IPA Quadrant Analysis of Perceived Tourism Image: Longwei Pass
Low importance-high performance
Keep stable
High importance-high performance
Keep up the good work
Low importance-low performance
Low priority
High importance-low performance
Concentrate here
Performance P
Importance I (%)
4.8
4.7
4.6
4.5
4.4
4.3
4.2
4.1
4.0
0
2
4
6
8
10
12
14
16
D2
B3
D4
C4
D1
C3
C1
A3
B2
A4
B1
C2
D3
A1
A2
B4
Space
A Basic spatial support
A1 Location and transportation
A2 Environmental sanitation
A3 Public service facilities
A4 Signage and tour guidance
Setting
B Recreation and consumption experience
B1 Food and snacks
B2 Photography and social media check-ins
B3 Visiting order
B4 Commercial consumption
Place
C Historical landscape cognition
C1 Traditional street-and-alley pattern
C2 Natural ecological landscape
C3 Historical and cultural relics
C4 Local folk life
Realm
D Emotional meaning and resonance
D1 Physical and mental experience
D2 Place identity
D3 Memory and spiritual sustenance
D4 Revisit and recommendation intention
