# Supplementary material for: Tourism image perception of living historical and cultural neighborhoods under the HSSPR framework: A comparative study of three cases in Yunnan, China
Source: PLoS One. 2026 Aug 14;21(8):e0355218. doi: 10.1371/journal.pone.0355218 (PMC13475901; doi:10.1371/journal.pone.0355218)
Supplement: Supplementary file 1 [file pone.0355218.s001.zip › Supporting Information/05_IPA_16_Element_Analysis_Results/IPA_redrawn_English_SCI_compact/Ming-Qing_Tonghai_Old_Town_IPA_EN_SCI_compact.pdf]

IPA Quadrant Analysis of Perceived Tourism Image: Ming-Qing Tonghai Old Town

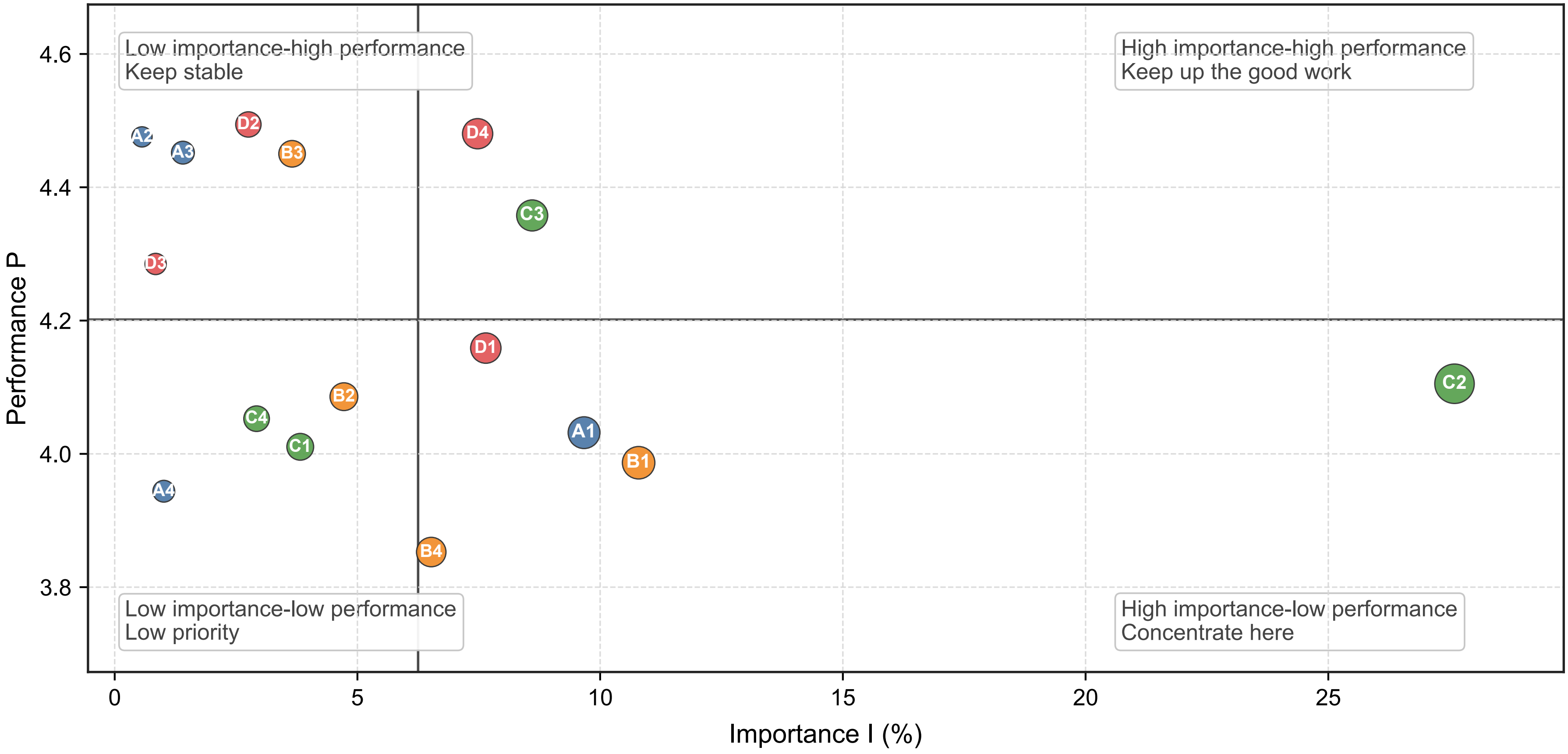

Space	Setting	Place	Realm
**A Basic spatial support**	**B Recreation and consumption experience**	**C Historical landscape cognition**	**D Emotional meaning and resonance**
A1 Location and transportation	B1 Food and snacks	C1 Traditional street-and-alley pattern	D1 Physical and mental experience
A2 Environmental sanitation	B2 Photography and social media check-ins	C2 Natural ecological landscape	D2 Place identity
A3 Public service facilities	B3 Visiting order	C3 Historical and cultural relics	D3 Memory and spiritual sustenance
A4 Signage and tour guidance	B4 Commercial consumption	C4 Local folk life	D4 Revisit and recommendation intention
