# Supplementary material for: Tourism image perception of living historical and cultural neighborhoods under the HSSPR framework: A comparative study of three cases in Yunnan, China
Source: PLoS One. 2026 Aug 14;21(8):e0355218. doi: 10.1371/journal.pone.0355218 (PMC13475901; doi:10.1371/journal.pone.0355218)

IPA Quadrant Analysis of Perceived Tourism Image: Santai Mountain Historic Area

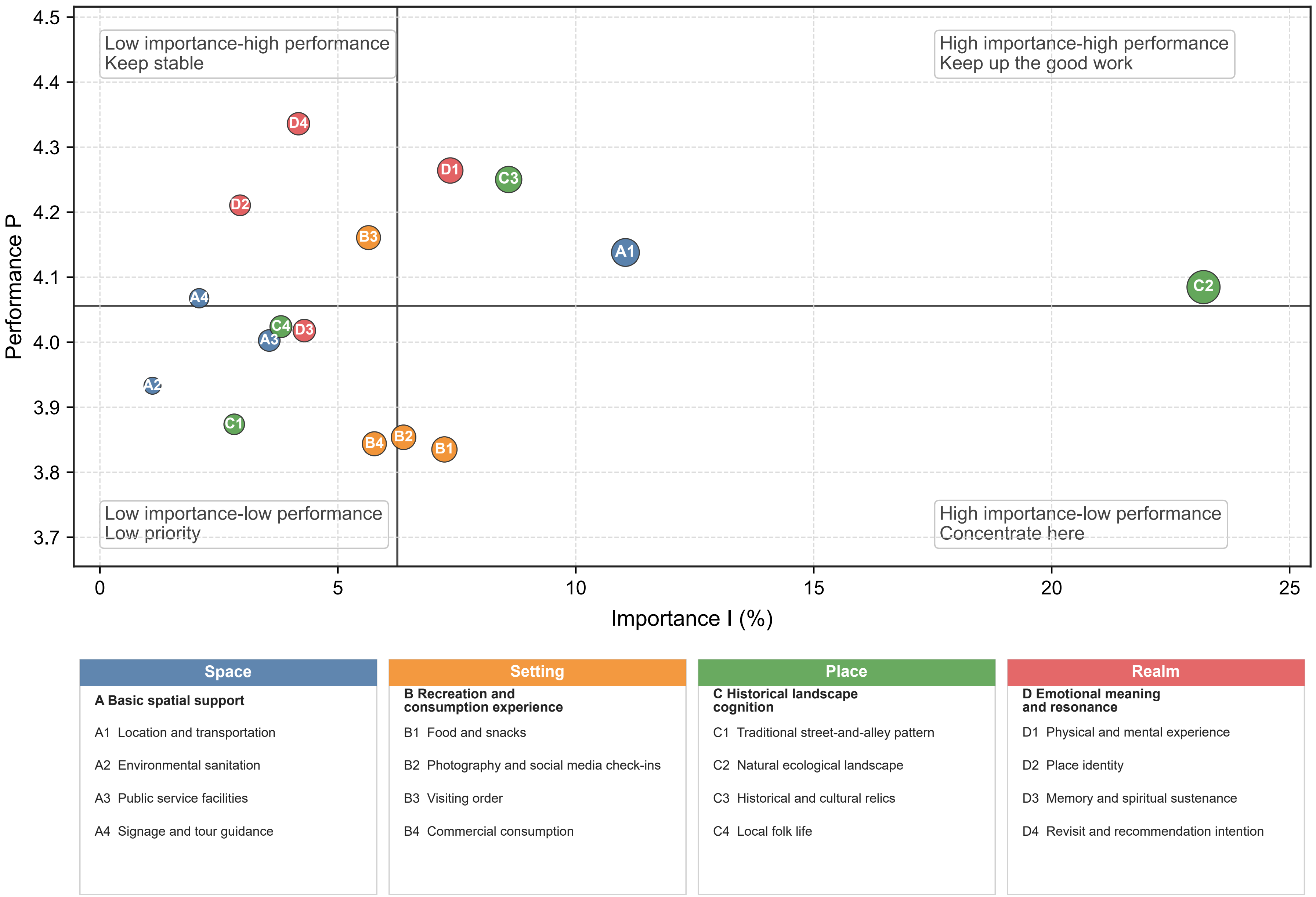

Supplement: Supplementary file 1 [file pone.0355218.s001.zip › Supporting Information/05_IPA_16_Element_Analysis_Results/IPA_redrawn_English_SCI_compact/Santai_Mountain_Historic_Area_IPA_EN_SCI_compact.pdf]
