# Supplementary material for: Tourism image perception of living historical and cultural neighborhoods under the HSSPR framework: A comparative study of three cases in Yunnan, China
Source: PLoS One. 2026 Aug 14;21(8):e0355218. doi: 10.1371/journal.pone.0355218 (PMC13475901; doi:10.1371/journal.pone.0355218)
Supplement: Supplementary file 1 [file pone.0355218.s001.zip › Supporting Information/06_Semantic_Network_Analysis/Semantic_Network_Outputs_EN/Longwei_Pass_Semantic_Network.pdf]

B

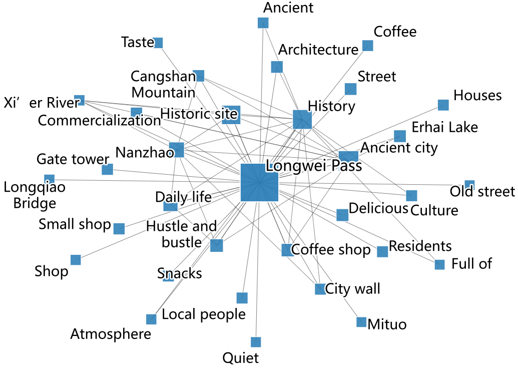
Ancient
Coffee
Taste
Architecture
Cangshan
Mountain
Street
Houses
Xi’ er River
History
Historic site
Commercialization
Erhai Lake
Ancient city
Nanzhao
Gate tower
Longwei Pass
Longqiao
Bridge
Old street
Daily life
Culture
Delicious
Small shop
Hustle and
bustle
Residents
Coffee shop
Full of
Shop
Snacks
City wall
Local people
Mituo
Atmosphere
Quiet
