# Supplementary material for: Tourism image perception of living historical and cultural neighborhoods under the HSSPR framework: A comparative study of three cases in Yunnan, China
Source: PLoS One. 2026 Aug 14;21(8):e0355218. doi: 10.1371/journal.pone.0355218 (PMC13475901; doi:10.1371/journal.pone.0355218)
Supplement: Supplementary file 1 [file pone.0355218.s001.zip › Supporting Information/06_Semantic_Network_Analysis/Semantic_Network_Outputs_EN/Ming-Qing_Tonghai_Old_Town_Semantic_Network.pdf]

D

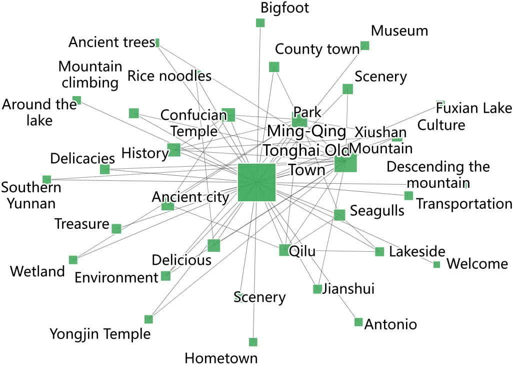
Bigfoot
Museum
Ancient trees
County town
Mountain climbing
Rice noodles
Scenery
Around the lake
Confucian Temple
Park
Fuxian Lake Culture
Ming-Qing Tonghai Old Town
Xiushan Mountain
History
Delicacies
Descending the mountain
Southern Yunnan
Ancient city
Transportation
Seagulls
Treasure
Qilu
Lakeside
Welcome
Wetland
Delicious
Environment
Jianshui
Scenery
Antonio
Yongjin Temple
Hometown
