# Supplementary material for: Tourism image perception of living historical and cultural neighborhoods under the HSSPR framework: A comparative study of three cases in Yunnan, China
Source: PLoS One. 2026 Aug 14;21(8):e0355218. doi: 10.1371/journal.pone.0355218 (PMC13475901; doi:10.1371/journal.pone.0355218)
Supplement: Supplementary file 1 [file pone.0355218.s001.zip › Supporting Information/06_Semantic_Network_Analysis/Semantic_Network_Outputs_EN/Santai_Mountain_Historic_Area_Semantic_Network.pdf]

C

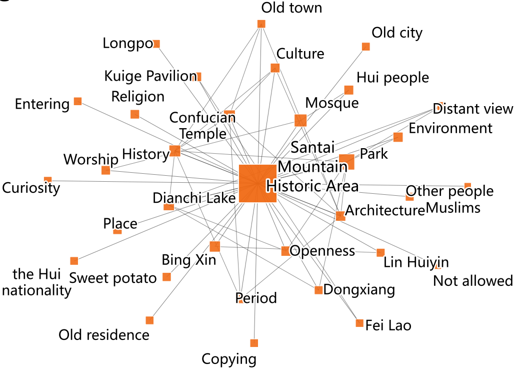

Old town
Old city
Longpo
Culture
Kuige Pavilion
Hui people
Entering
Religion
Mosque
Distant view
Confucian
Temple
Environment
Santai
Mountain
Historic Area
Park
History
Worship
Curiosity
Dianchi Lake
Other people
Muslims
Architecture
Place
Openness
Bing Xin
Lin Huiyin
the Hui
nationality
Sweet potato
Not allowed
Period
Dongxiang
Old residence
Fei Lao
Copying
