# Supplementary material for: Tourism image perception of living historical and cultural neighborhoods under the HSSPR framework: A comparative study of three cases in Yunnan, China
Source: PLoS One. 2026 Aug 14;21(8):e0355218. doi: 10.1371/journal.pone.0355218 (PMC13475901; doi:10.1371/journal.pone.0355218)
Supplement: Supplementary file 1 [file pone.0355218.s001.zip › Supporting Information/06_Semantic_Network_Analysis/Semantic_Network_Outputs_EN/Three_Sites_Integrated_Semantic_Network.pdf]

A

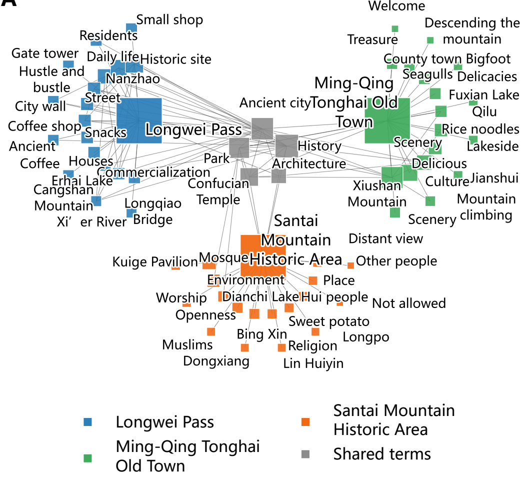

Small shop
Residents
Gate tower
Daily life
Historic site
Hustle and bustle
Nanzhao
Street
City wall
Coffee shop
Snacks
Longwei Pass
Ancient
Coffee
Houses
Erhai Lake
Commercialization
Cangshan Mountain
Longqiao Bridge
Xi' er River
Welcome
Descending the mountain
Treasure
County town
Bigfoot
Seagulls
Delicacies
Ming-Qing Tonghai Old Town
Fuxian Lake
Qilu
Rice noodles
Lakeside
Scenery
Delicious
Culture
Jianshui
Xiushan Mountain
Mountain climbing
Scenery
Ancient city
History
Park
Architecture
Confucian Temple
Santai Mountain Historic Area
Distant view
Kuige Pavilion
Mosque
Other people
Environment
Place
Worship
Dianchi Lake
Hui people
Not allowed
Openness
Sweet potato
Bing Xin
Longpo
Muslims
Religion
Dongxiang
Lin Huiyin
Longwei Pass
Ming-Qing Tonghai Old Town
Santai Mountain Historic Area
Shared terms
